# Inhibition of androgen/AR signaling inhibits diethylnitrosamine (DEN) induced tumour initiation and remodels liver immune cell networks

**DOI:** 10.1038/s41598-021-82252-x

**Published:** 2021-02-11

**Authors:** Timothy H. Helms, Riley D. Mullins, Jennifer M. Thomas-Ahner, Samuel K. Kulp, Moray J. Campbell, Fabienne Lucas, Nathan Schmidt, Dana M. LeMoine, Surafel Getaneh, Zhiliang Xie, Mitch A. Phelps, Steven K. Clinton, Christopher C. Coss

**Affiliations:** 1 Department of Veterinary Biosciences, The Ohio State University, Columbus, OH 43210 USA; 2 The Comprehensive Cancer Center, The Ohio State University, Columbus, OH 43210 USA; 3 Division of Pharmaceutics and Pharmacology, College of Pharmacy, The Ohio State University, Columbus, OH 43210 USA; 4 Biomedical Informatics Shared Resource, The Ohio State University, Columbus, OH 43210 USA; 5 Division of Hematology, Department of Medicine, The Ohio State University, Columbus, OH 43210 USA; 6 University Laboratory Animal Resources, The Ohio State University, Columbus, OH 43210 USA; 7 Division of Medical Oncology, Department of Internal Medicine, The Ohio State University, Columbus, OH 43210 USA

**Keywords:** Cancer models, Hepatocellular carcinoma, Steroid hormones

## Abstract

A promotional role for androgen receptor (AR) signaling in hepatocellular carcinogenesis is emerging. In pre-clinical models, including diethylnitrosamine- (DEN-) induced hepatocellular carcinoma (HCC), anti-androgen therapies delay hepatocarcinogenesis. However, pharmacologic anti-androgen therapy in advanced HCC patients fails, suggesting that AR plays a role in HCC onset. This study aims to characterize AR expression and function throughout DEN-induced liver inflammation and carcinogenesis and evaluate the efficacy of prophylactic AR antagonism to prevent hepatocarcinogenesis. We demonstrate that pharmacologic AR antagonism with enzalutamide inhibits hepatocellular carcinogenesis. With enzalutamide treatment, we observe decreased CYP2E1 expression, reducing DEN-induced hepatocyte death and DNA ethyl-adducts. AR protein expression analyses show that DEN causes an initial upregulation of AR in portal fibroblasts and leukocytes, but not hepatocytes, suggesting that hepatocyte-autonomous AR signaling is not essential for DEN-induced carcinogenesis. Ablating androgen signaling by surgical castration reduced pre-carcinogen Kupffer cell populations but did not alter DEN-mediated immune cell recruitment nor AR expression. In this study, we identified that anti-androgen interventions modulate mutagenic DNA adducts, tumour initiation, and immune cell composition. Additionally, we find that AR expression in hepatocytes is not present during nor required for early DEN-mediated carcinogenesis.

## Introduction

The androgen receptor (AR) is an enticing therapeutic target in hepatocellular carcinoma (HCC). This belief is founded upon the recognition of a marked sexual dimorphism in HCC risk, with men three to eight times more likely than women to develop or die from the disease^1^. HCC emerges in the presence of chronic liver disease and the sexual dimorphism is evident regardless of the underlying aetiology including viral hepatitis, alcoholic liver disease, non-alcoholic fatty liver disease, and chronic aflatoxicosis. Intriguingly, the sexual dimorphism is consistent across species as multiple rodent models of HCC also exhibit a striking male predisposition^2–4^. Molecular evidence further supports a pro-carcinogenic role for the AR-signaling in HCC. Increased AR expression in both human and rodent HCC tumours has been reported^5–9^. More recent analyses implicate AR signaling may activate a variety of critical cancer-associated pathways in the neoplastic liver, including suppression of DNA repair, enhancing cancer cell metabolic activity and division, activation of known oncogenes, and promotion of liver cancer cell immortalization^9–13^.

In rodent models, anti-androgen interventions including surgical castration, heritable mutations of the AR, and liver-specific AR-knockout reliably inhibit hepatocarcinogenesis, implicating a neoplasia-promoting capacity for AR signaling^9,14,15^. The success of anti-androgen therapies in pre-clinical carcinogenesis models has yet to translate into humans as pharmacologic targeting of the AR-signaling axis consistently fails when studied in late-stage HCC^16,17^. These findings support a hypothesis that inhibition of the liver carcinogenesis cascade may require early and prolonged intervention of the AR-signaling axis. In both carcinogen- and viral transgene-induced models, surgical castration as a therapeutic intervention demonstrates a precipitous decline in anti-carcinogenic activity with delayed orchiectomy^3,4^. Thus, disruption of androgen signaling early in the carcinogenic cascade, perhaps with a chemoprevention strategy, warrants consideration.

Our understanding of AR-signaling in early hepatocarcinogenesis—where its tumour-inhibiting outcomes are maximized in HCC animal models—remain poorly understood. Given the current body of evidence, we hypothesized that targeted pharmacologic intervention at the AR signaling axis would be most effective early in hepatocarcinogenesis. To test this hypothesis—and to interrogate mechanisms of action potentially explaining the efficacy of early antagonism of the AR signaling axis in HCC—we treated (i.e. before carcinogenic challenge) adult rats with enzalutamide (ENZ), a potent AR antagonist, in a diethylnitrosamine (DEN)-induced model of hepatocellular carcinogenesis.

## Results

### Antagonism of the AR inhibits DEN hepatocarcinogenesis

#### Study design

**Figure 1:**
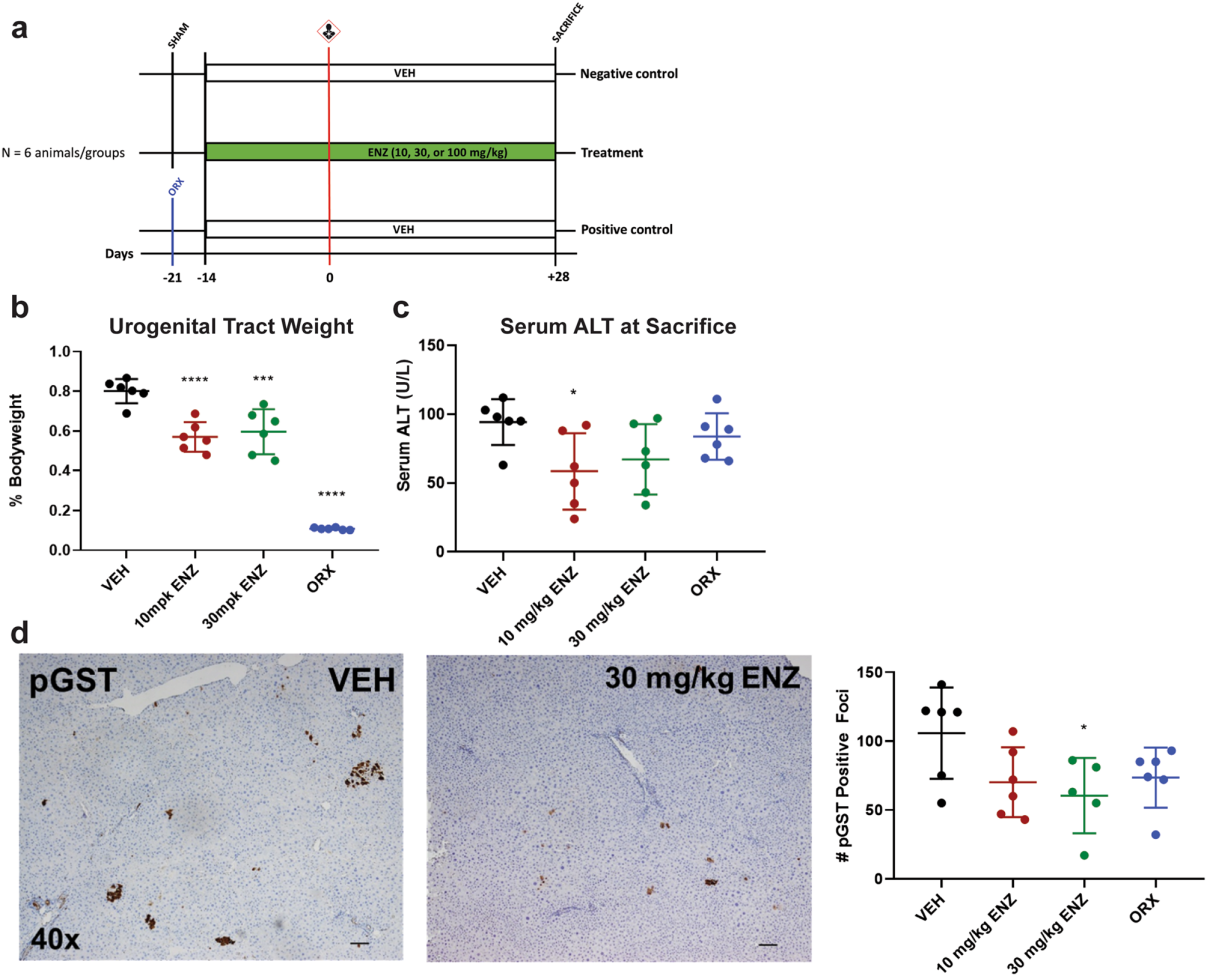
Low dose, preventive ENZ inhibits DEN-induced hepatocellular carcinogenesis. (**a**) Study Design—simplified timeline highlighting preventive anti-androgen interventions. Carcinogen challenge consisting of the described DEN/PH/DEN procedure is represented by carcinogen pictogram. SHAM, sham castration; ORX, castration. Enzalutamide (ENZ) groups received 10, 30, or 100 mg/kg and control groups received vehicle (VEH), all once daily by oral gavage. Carcinogen-challenged, preventive ORX served as anti-androgen phenotype positive controls; carcinogen-challenged, SHAM rats served as anti-androgen phenotype negative controls. (**b**) Low-dose ENZ maintained an anti-androgen effect on bodyweight-normalized urogenital tract weights. *** *p* < 0.0005; ****p < 0.0001 (vs. VEH), one-way ANOVA followed by Dunnett’s multiple comparisons. (**c**) ENZ at 10 mg/kg reduced liver damage marker, serum ALT(U/L). *p = 0.0298 (10 mg/kg ENZ vs. VEH), one-way ANOVA followed by Dunnett’s multiple comparisons. (**d**) ENZ at 30 mg/kg reduced the number of pGST-positive, preneoplastic foci. Left, Representative photomicrographs (40 × total magnification) of IHC for pGST for VEH (left) and 30 mg/kg ENZ (right) treatment groups. Right, Comparison of the total number of pGST-positive foci between treatment groups. *p = 0.033 (30 mg/kg vs. VEH), one-way ANOVA followed by Dunnett’s Multiple Comparisons. All data represented as mean ± standard deviation.

To test the hypothesis that competitive antagonism of AR signaling inhibits hepatocellular carcinogenesis, we administered ENZ orally once daily to male rats in three separate dose groups (Fig. 1a). To ensure steady-state concentrations of drug were achieved, doses were administered for two weeks prior to carcinogenic challenge consisting of two-thirds partial hepatectomy (PH) and IP injections of 50 mg/kg DEN at 24 h prior to and following the PH procedure. DEN dosage and timing were modeled after a previous report of DEN carcinogenesis in rats examining similar outcomes^18^. ENZ dosages were based on a systemic exposure-matched therapeutic equivalent for patients receiving treatment for castrate resistant prostate cancer (100 mg/kg) and half-log equivalents thereof, 30 mg/kg and 10 mg/kg^19^. To ensure maximal AR antagonism, both DEN challenges were timed to occur at approximately within-dose maximal ENZ plasma concentrations (T_max_). Vehicle-treated SHAM animals receiving DEN/PH alone served as untreated controls while animals orchiectomized (ORX) three weeks prior to carcinogen challenge served as anti-androgen phenotype positive controls. We observed mortality at the time of carcinogen challenge within the 100 mg/kg group that left only two animals surviving to the end of study; this treatment group was thus excluded from follow-up analysis.

#### Low-dose ENZ maintains anti-androgen efficacy

To ensure that low-dose ENZ as administered exhibits anti-androgen efficacy in the rat, the androgen-dependent tissues of the lower urogenital tract (prostate, coagulating gland, seminal vesicles, and urinary bladder) were harvested and examined (Fig. 1b). Consistent with pharmacologic antagonism of the AR, we observed a decrease in urogenital tract weight with ENZ across all doses^19^. Surgical castration resulted in marked atrophy of the accessory sex glands.

#### Low dose ENZ reduces serum ALT 4 weeks after carcinogen challenge

In rodents, ENZ is primarily cleared by hepatic metabolism^19^. To ensure therapeutic tolerability in an experimental design with considerable physiologic challenge to the liver, we assessed study morbidity, mortality, gross pathology and liver damage biomarkers. All rats on low dose (10, 30 mg/kg) ENZ tolerated carcinogenic challenge and survived to end of study. Livers collected at necropsy were enlarged compared to age-standardized controls, a phenotype consistent with both the addition of DEN carcinogen and long-term ENZ administration^9,19,20^. There was no appreciable difference in gross appearance of the livers collected at sacrifice, and there was no impedance of hepatic regeneration with anti-androgen therapies as determined by liver weights normalized to bodyweight (Supplementary Fig. S1 online). Serum ALT is a common biomarker for acute hepatocellular necrosis; its value correlates to downstream carcinogenesis in the DEN model^21^. We observed no significant differences in serum ALT at the time of carcinogenic challenge (Supplementary Fig. S1 online). However, there was a statistically significant reduction in serum ALT at sacrifice with 10 mg/kg ENZ administration (*p = 0.0298, Fig. 1c).

#### ENZ inhibits hepatocellular carcinogenesis

To evaluate carcinogenic outcomes, we performed IHC using the pre-neoplastic marker pGST, a well-characterized predictor of carcinogenesis for a variety of carcinogens, including DEN^22^. pGST-positive initiated hepatocytes and foci of hepatocytes exhibited a random, multifocal distribution throughout the hepatic parenchyma. Four weeks following carcinogen challenge, we observed a trending decrease in pGST-positive focus number with ENZ administration, regardless of intervention, with a 30 mg/kg dose yielding a statistically significant reduction in the total number of pGST-positive foci compared to untreated controls (*p* = 0.033, Fig. 1d).

### Preventive ENZ inhibits DEN carcinogenesis in a model-specific manner

**Figure 2:**
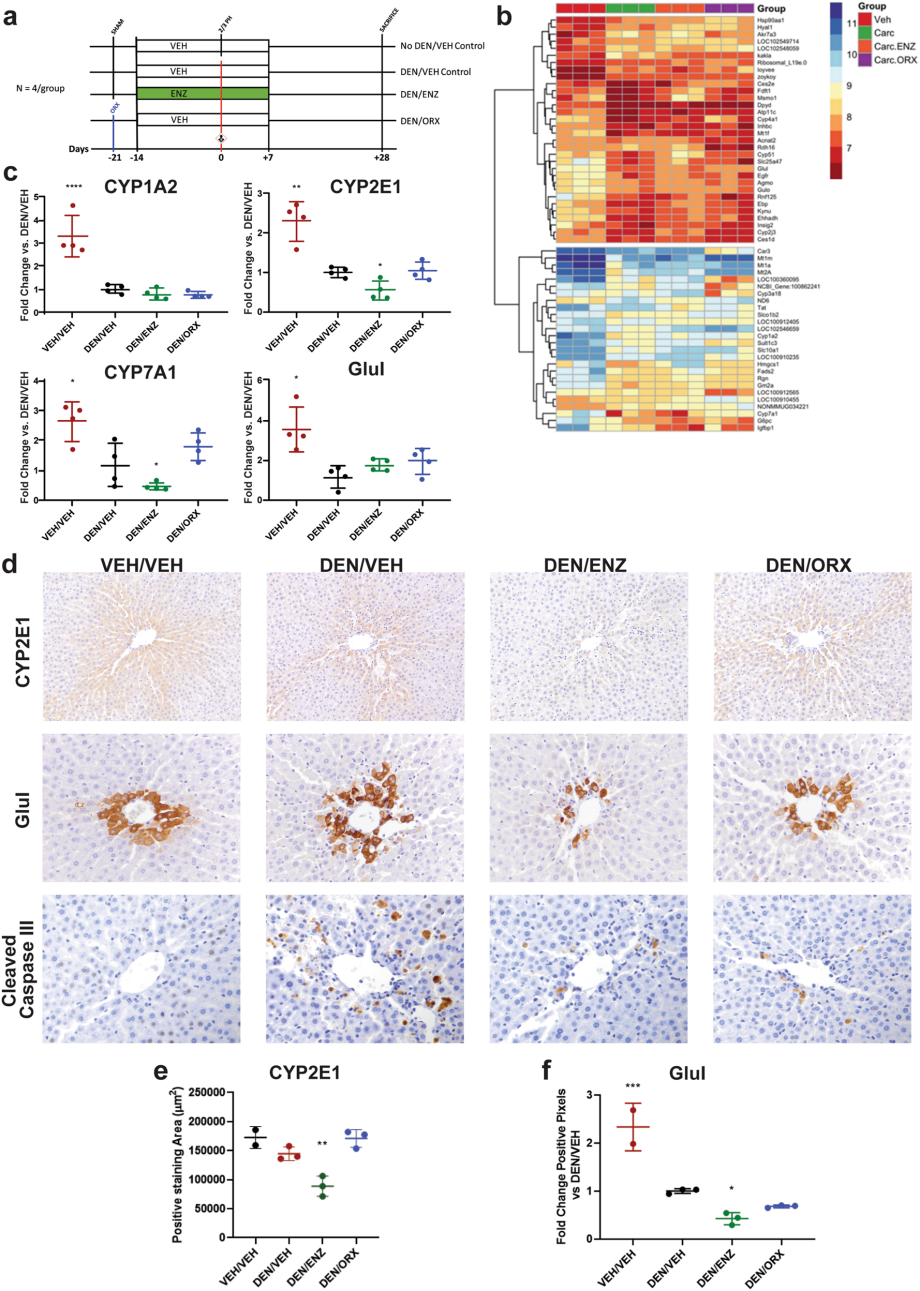
Preventive anti-androgen treatments inhibit DEN carcinogenesis in a model specific manner. (**a**) Study Design—simplified timeline highlighting preventive anti-androgen interventions for follow-up study. ENZ = 30 mg/kg daily oral gavage doses. Note addition of non-DEN, VEH-treated, PH only control group. (**b**) Heatmap highlighting gene expression (row) by animal (column) within treatment group (column color). Red = downregulation; Blue = upregulation. (**c**) qRT-PCR confirmation of expression of Wnt/β-catenin targets CYP1A2, CYP2E1, CYP7A1, Glul. Preventive ENZ suppresses CYP2E1 and CYP7A1 expression. (**d**) Zonal distribution of CYP2E1 (200x) and Glul (400x) is reduced by preventive ENZ; ENZ and ORX suppress DEN induction of CCIII (400x). (**e**) ENZ reduces CYP2E1 positive staining area (quantification of 2D CYP2E1). (**f**) ENZ reduces Glul expression (quantification of 2D Glul) Quantitative, positive pixel analysis supports this conclusion. All analyses are One-Way ANOVA, Dunnett’s Multiple comparisons (vs. DEN/VEH): *p < 0.05, **p < 0.01, ***p < 0.001, ****p < 0.0001. All data represented as mean ± standard deviation.

Previous reports concerning AR signaling in the context of in vivo DEN carcinogenesis investigated the role of AR signaling when neoplasia is established. Given our observation that preventive pharmacologic and surgical inhibition of the AR signaling axis inhibits hepatocellular carcinogenesis, we were curious to explore potential mechanistic hypotheses clarifying AR’s function earlier at the time of carcinogen challenge. To accomplish this task, we performed a follow-up experiment of similar design with the addition of a non-DEN challenged/no intervention group as a PH-only control (Fig. 2a). We performed mRNA microarray on livers collected at PH, 24-h after DEN challenge, designed to capture immediate treatment-based differences in the hepatic AR’s transcriptome in response to DEN carcinogen.

#### ENZ and ORX differentially modulate hepatic gene expression in response to DEN

Principal component analysis and hierarchical clustering identified a treatment-based segregation of gene expression profiles with unchallenged, carcinogen-challenged, and antiandrogen intervention groups forming three distinct clusters (Supplementary Fig. S2 online). To gain insight into the functional annotation of our differentially expressed genes, we performed gene-set enrichment analysis comparing the intervention groups to the carcinogen-challenged control group (Supplementary Fig. S2 online). With challenge only, we observed a marked downregulation of numerous hepatic metabolic pathways including the following: four gene sets associated with lipid biosynthesis and metabolism, four associated with xenobiotic metabolism pathways, and three associated with steroid and sterol metabolic pathways. Preventive ENZ caused a strong, selective rescue of fatty acid degradation and cholesterol biosynthesis genes with thirteen of the lowest p-value gene sets highlighting upregulated cholesterol biosynthesis and lipid oxidation pathways. Intriguingly, despite their close hierarchical clustering, we observed no overlap in movement of genes expressed between anti-androgen treatment (ENZ) and hormone ablation (ORX) (Supplementary Fig. S2 online). As favored as ENZ intervention was toward upregulation of cholesterol metabolism and biosynthesis, ORX yielded an equally strong upregulation of pathways associated with cellular responses to oxidative stressors, specifically zinc, transition metal and inorganic ions.

#### ENZ suppresses hepatic β-catenin targets, CYP2E1, and Glutamine Synthetase (Glul)

Cholesterol metabolism/fatty acid degradation, bile acid synthesis, and detoxification are all confined processes occurring within specific compartments of the larger hepatic lobule, a phenomenon known as hepatic metabolic zonation^23^. Cholesterol biosynthesis pathways are limited to the periportal hepatocyte while CYP detoxification and bile acid synthesis pathways are restricted to the centrilobular hepatocyte. A major regulator of hepatic metabolic zonation is the Wnt/β-catenin signaling pathway which establishes the centrilobular hepatocyte phenotype^23^. Several known β-catenin target genes exhibited differential expression between our various treatment groups including: Glul, CYP1A2, epidermal growth factor receptor (EGFR), regucalcin (Rgn), and CYP7A1 (Fig. 2b)^24,25^. Given our treatment groups’ apparent differential modulation of zonally regulated phenomena (ENZ favoring periportal fatty acid degradation pathways; ORX favoring upregulation of perivenous detoxification and bile acid synthesis pathways), we hypothesized that our preventive anti-androgen therapies may be differentially influencing hepatic Wnt/β-catenin-mediated metabolic zonation in the face of DEN challenge. To test this hypothesis, we evaluated downstream targets of Wnt/β-catenin activity using a candidate gene approach, namely: CYP1A2, CYP2E1, CYP7A1, and Glul (Fig. 2c). Consistent with our microarray, qRT-PCR confirmed a robust downregulation of CYP1A2, CYP7A1, and Glul subsequent to a single DEN injection (Fig. 2b,c). Despite not exhibiting differential expression in the microarray analyses, CYP2E1 followed a similar expression pattern to other Wnt/β-catenin targets. Preventive castration exhibited no differences in gene expression compared to DEN-challenged, VEH-treated controls suggesting no ORX-based modification of metabolic zonation. Preventive ENZ, on the other hand, reduced CYP7A1 and CYP2E1 transcript levels compared to DEN-treated controls.

To further analyze potential ENZ-mediated suppression of Wnt/β-catenin activity, we interrogated the expression and spatial distribution of CYP2E1 and Glul (Fig. 2d). We focused on these two enzymes as they are among the best characterized downstream targets of β-catenin activity in the liver with zonal distribution around central veins ^23,24,26^. We performed IHC for CYP2E1 and Glul on liver tissues collected at PH hypothesizing that ENZ restricts the distribution of CYP2E1 and Glul expression. Consistent with our hypothesis, we observed a reduction and restriction of CYP2E1 and Glul expression with ENZ treatment compared to DEN/VEH-treated animals, a finding supported quantitatively through overall area of expression and positive pixel analysis for CYP2E1 and Glul, respectively (Fig. 2e,f) and CYP2E1 protein levels measured by western blot (Supplementary Fig. S2 online). Together, these findings support that ENZ inhibits hepatic β-catenin activity and disrupts metabolic zonation of the hepatic lobule.

#### ENZ-induced suppression of CYP2E1 inhibits DEN-induced hepatocyte death and carcinogenesis

CYP2E1 is the phase I enzyme primarily responsible for DEN oxidation and bio-activation^﻿26﻿,27^. DEN activation leads to free-radical induced oxidative injury sometimes leading to induced cell death, or DNA adduct formation causing mutations through mismatch misrepair^28^. Given ENZ’s suppressive effects on CYP2E1 expression, we hypothesized that ENZ would reduce DEN bioconversion resulting in decreased DEN-induced cell death and mutagenic adducts. To test this hypothesis, we performed IHC for cleaved caspase III (CCIII) to evaluate DEN-induced hepatocyte death, and quantified DEN-adduct O_2_-ethyl-thymidine in DNA extracted from liver samples collected at PH. This particular ethyl-adduct was chosen for its documented persistence and mutagenicity^29^. Consistent with this hypothesis, preventive ENZ reduced the number of CCIII positive hepatocytes (Fig. 2d) and quantity of O_2_-ethyl-thymidine adducts formed in DEN-treated animals (Supplementary Fig. S2). Intriguingly, castration yielded a similar effect despite no observed suppression of CYP2E1 expression further implicating a distinct mechanism of action in ENZ-treated animals.

### Characterization of the hepatic AR expression in DEN carcinogenesis

In light of our findings supporting that ENZ and ORX inhibit carcinogenesis at its earliest, initial phases of DNA adduct formation, we were curious to investigate the expression and distribution of the hepatic AR in early hepatocarcinogenesis. There is ample evidence highlighting AR upregulation in HCC tumors^7–9,29–31^. However, most of this data is derived from tissue homogenate-based ligand binding assays that lack cell-type specificity and there is no clear consensus on the immunohistochemical distribution of hepatic AR^7,9,30,31^. Furthermore, consistent evidence of where the AR is expressed or how its expression changes during carcinogenesis is lacking. Given our model, we recognized the opportunity to better understand hepatic AR expression in the early stages of liver carcinogenesis.

#### AR is upregulated early in hepatocarcinogenesis

It is well established that DEN-induced pGST-positive foci, or early, pre-neoplastic lesions, grow and develop into hepatocellular adenomas and carcinomas. We hypothesized that AR is upregulated early in hepatocarcinogenesis and that increased expression localizes to early, pre-neoplastic lesions.

**Figure 3:**
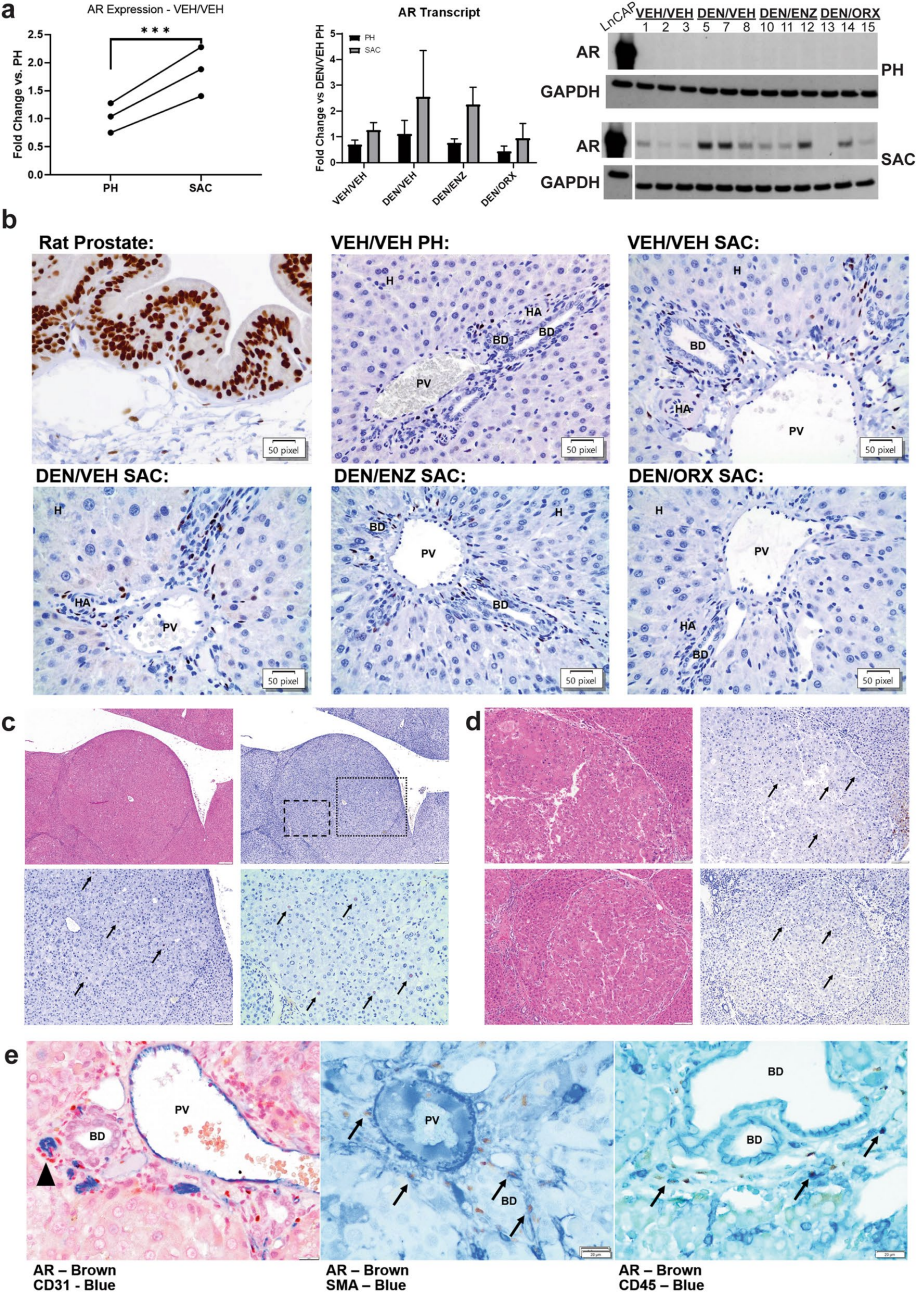
Characterization of the hepatic AR in DEN carcinogenesis. (**a**) Left: qRT-PCR comparison of AR expression in non-DEN challenged liver collected at partial hepatectomy (PH) versus sacrifice (SAC). AR transcript expression at SAC is higher (Paired T-test, ***p = 0.0005) implicating a PH-dependent increase in AR signaling. Middle: qRT-PCR comparison of hepatic AR expression at PH and SAC based on DEN challenge and intervention. Significant sources of variation include PH (*p* = 0.0002) and within subject (*p* = 0.0034) effects. Data represented as mean ± standard deviation. Right: Western blot for AR at PH and SAC within subjects (Samples from the same animals at PH and SAC are represented in the same lane). GAPDH = reference protein. LNCaP cell lysate was used as positive control. Individual lanes are labelled with each sample’s identification number, three samples per experimental group. Consistent with transcript data, AR is upregulated with DEN challenge at SAC. Full-length blots are presented in Supplementary Fig. S5 online. (**b**) AR expression is limited to the portal triad in early DEN carcinogenesis. Top Left—positive control, rat prostate, note strong positive nuclear immunoperoxidase staining for AR in glandular epithelium and stromal cells. Remaining Panels—Liver: AR expression is limited to the portal triad/periportal area. Strong AR staining is in the nuclei of spindle to round-shaped nuclei lining bile ducts (BD) and portal vein (PV) and hepatic arteriole (HA). Parenchymal hepatocytes (H) lack AR staining. There is rare, light nuclear staining of smooth muscle walls of the HA and limiting plate hepatocytes. There is no change in the distribution of AR expression with DEN or intervention. (**c**) AR expression is scarce in DEN-induced adenomas. Top Left—H&E, photomicrograph of AR-positive adenoma. Top Right—IHC for AR of same adenoma. Bottom Left—200 × total magnification inset of dotted region from top left panel. Bottom Right—400 × total magnification inset of dashed region from top left panel. AR immunoperoxidase-positive nuclei of neoplastic hepatocytes are highlighted with arrows. (**d**) AR expression is scarce in DEN-induced carcinomas. Left—Photomicrographs of representative H&E-stained sections of two AR-positive carcinomas. Right—Photomicrographs of AR IHC in the same carcinomas which again highlight rare to occasional nuclear AR expression in neoplastic hepatocytes (arrows). Cytoplasmic staining is non-specific edge artefact as determined by rabbit IgG isotype, and no primary antibody controls (data not shown). (**e**) Left—AR-positive nuclei (brown) do not co-localize with cytoplasmic CD31 (blue) but are often seen around CD31-positive vascular structures (arrowhead; 600 × total magnification). Middle—AR-positive nuclei are frequently surrounded by SMA-positive cytoplasmic staining (blue, arrows; 600 × total magnification). Right—AR-positive nuclei are often surrounded by CD45-positive staining cytoplasm (blue, arrows; 600 × total magnification).

To evaluate the effects of PH and recovery on hepatic AR transcript expression, we compared liver samples collected at PH and 28 days later at sacrifice within vehicle-treated, non-DEN-challenged animals. We observed an approximate doubling of hepatic AR transcript at sacrifice, implying a time or PH-dependent induction of the hepatic AR over the four weeks between carcinogen challenge and sacrifice (Fig. 3a). DEN treatment, both at the time of PH and sacrifice, increased mean AR transcript levels compared to vehicle-treatment. Consistent with our transcript findings, we observed a similar expression pattern at sacrifice on Western blot analysis (Fig. 3a). Two-way, repeated measures ANOVA revealed that much of the variance in transcript observed at the two evaluated time-points could be attributed to within subject (42.18%) or the aforementioned PH-dependent (26.2%) effects. Preventive anti-androgen interventions did not account for a statistically significant effect on hepatic AR transcript expression.

#### The pre-neoplastic hepatocyte does not express AR

Contrary to our hypothesis and the reports of others, AR was undetectable in the broader parenchyma, including hepatocytes, in IHC analyses (Fig. 3b)^9^. Instead, AR expression localized to the portal triad with infrequent, nuclear immunoperoxidase staining of the smooth muscle of hepatic arterioles; more common, robust nuclear staining of spindle cells lining bile ducts and portal vasculature; and staining of round cell nuclei residing independently within the portal interstitium. Consistent with transcript and Western blot analyses, an increase in AR expression by IHC was apparent in samples collected at SAC compared to those at PH. Importantly, neither anti-androgen intervention nor time (i.e. PH vs. SAC) altered the distribution of AR expression within the liver. In each case, the distribution pattern of hepatic AR expression was limited to the periportal region of the hepatic lobule, and there was no overlap in expression pattern when compared to the multifocal, scattered hepatocellular distribution of pGST.

#### Sparse AR upregulation in DEN-induced hepatocellular neoplasms

Given the absence of the AR from the parenchymal and pre-neoplastic hepatocyte in the rat model, we were curious to evaluate AR expression in DEN-induced HCC lesions. To this end, intact male rats were subjected to the DEN/PH challenge, allowed to recover without intervention for four weeks, and then administered weekly IP injections of DEN up to 18 weeks post-PH to accelerate carcinogenesis (Supplementary Fig. S3 online). Livers collected at 10, 14, and 18 weeks post-carcinogen challenge exhibited progressive fibrosis with corresponding gross and histologic characteristics including necrosis, bridging portal fibrosis, biliary hyperplasia, and nodular regeneration (Supplementary Fig. S3 online). AR expression in fibrotic livers was consistent with our prior observations and was confined to the periportal areas as well as extending along areas of bridging portal fibrosis and biliary hyperplasia. Hepatocytes were negative for AR (Supplementary Fig. S3 online). Later in the time course, we observed small, poorly circumscribed clusters of hepatocytes consistent with foci of hepatocellular alteration (Supplementary Fig. S3 online), compressive adenomas, and occasional carcinomas. The majority of neoplasms were devoid of AR staining (data not shown). However, some tumors had occasional, random but consistent immunoperoxidase-positive nuclear AR staining within neoplastic hepatocytes (Fig. 3c,d).

#### Hepatic AR co-expresses with CD45 and smooth muscle actin

To better identify AR-expressing cells within the portal triad, we performed dual chromogen IHC co-stains on fibrotic rat livers. Given their location and distribution, our differentials for AR-positive cells were portal fibroblasts, immune cells, and vascular endothelial cells. We assessed the co-localization of nuclear AR-positive signal with membranous and cytoplasmic markers including: CD45 as a broad immune cell marker, CD31 for endothelium, and smooth muscle actin (SMA) for activated portal fibroblasts (Fig. 3e). AR-positive cells were not of endothelial origin as cytoplasmic CD31 did not co-localize with nuclear AR expression. There was a strong correlation, however, where AR positive nuclei were found surrounding CD31-positive vascular structures. Both SMA and CD45 exhibited cytoplasmic co-localization with nuclear AR staining, suggesting large portions of total hepatic AR expression can be attributed to activated portal fibroblasts and inflammatory cells.

#### Impact of DEN challenge and hormone status on hepatic immune cell populations

**Figure 4:**
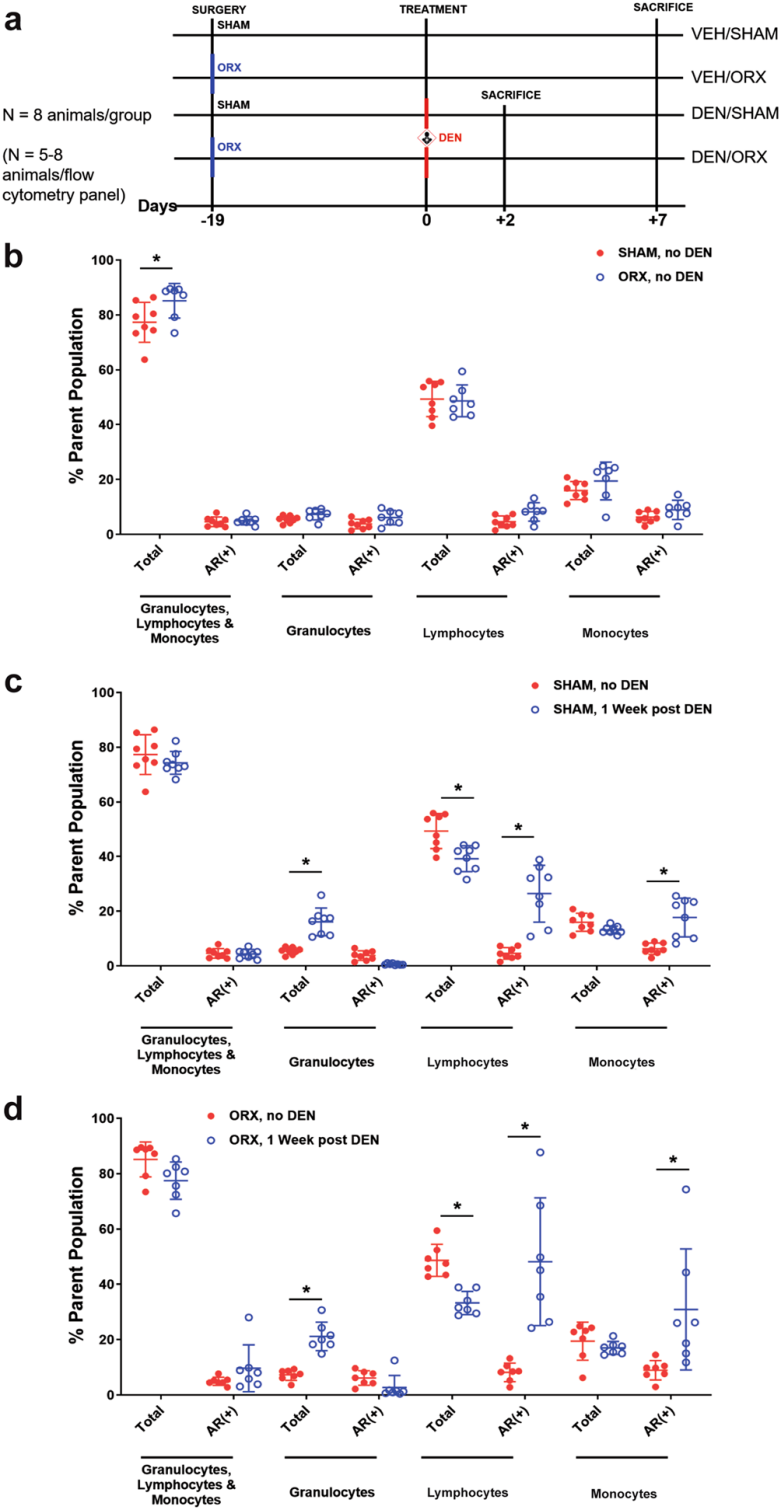
DEN increases proportion of AR+ lymphocytes and monocytes in SHAM and ORX. Liver immune cell populations were analyzed by flow cytometry as described in Materials and Methods and represented as percent of parent population and pre-gated on CD45+ and living cells. Positive cells were defined by fluorescence minus one (FMO) controls (See Supplementary Fig. S4 online for gating strategy). (**a**) Simplified experimental timeline. Surgery was performed on 8 mice per group 19 days prior to treatment with IP VEH or DEN (50 mg/kg). DEN mice were sacrificed at 2 and 7 days following treatment, and VEH mice were sacrificed at 7 days following treatment. (**b**) ORX increased the percentage of total CD45+ cells (*p < 0.05, Sidak's multiple comparisons). (**c**) DEN challenge in SHAM mice increased the proportions of granulocytes, AR+ lymphocytes and monocytes and decreased that of lymphocytes at 1 week after DEN challenge (*p < 0.05, Sidak's multiple comparisons). (**d**) The changes in ORX mice were similar to those in SHAM mice in response to DEN challenge (*p < 0.05, Sidak's multiple comparisons). All data represented as mean ± standard deviation.

Given AR’s absence from rat liver hepatocytes and preliminary IHC evidence suggesting expression in hepatic immune (CD45+) cells, we hypothesized that AR-positive immune cell recruitment could contribute to the increased AR expression described and observed in DEN carcinogenesis. To test this, we challenged SHAM and ORX mice with VEH or 50 mg/kg IP DEN and evaluated their hepatic immune cell populations by flow cytometry one-week after DEN exposure (Fig. 4a). Although our previous experiments utilized rats due to the abundance of DEN carcinogenesis work done in this species, C57BL/6 mice were used for this experiment due to the improved availability of flow cytometry anti-mouse antibodies, suitability for follow-up studies implementing xenograft or genetically modified models, and a robust literature on DEN carcinogenesis in mouse.

#### DEN increases AR expression in hepatic monocytes and lymphocytes 1-week after challenge

To test our hypothesis, we first assessed the fraction of AR+ cells within hepatic immune cell subsets one week after VEH or DEN challenge using side scatter profiles to differentiate lymphocytes, granulocytes, and monocytes. The percent of total immune cells (CD45+) was slightly increased in ORX mice in the absence of DEN challenge (Fig. 4b). That said, hormone status did not significantly modulate the proportions of immune cell subsets nor the percent of AR+ cells, with the latter finding in broad agreement with AR expression from the analyses in rat. Despite ablation of circulating androgens in the ORX group, there were similar increases in the percent of AR+ lymphocytes and monocytes in both SHAM (Fig. 4c) and ORX (Fig. 4d) mice 1 week after DEN administration.

#### ORX modifies baseline hepatic immune cell populations, does not alter DEN inflammatory cell response

**Figure 5:**
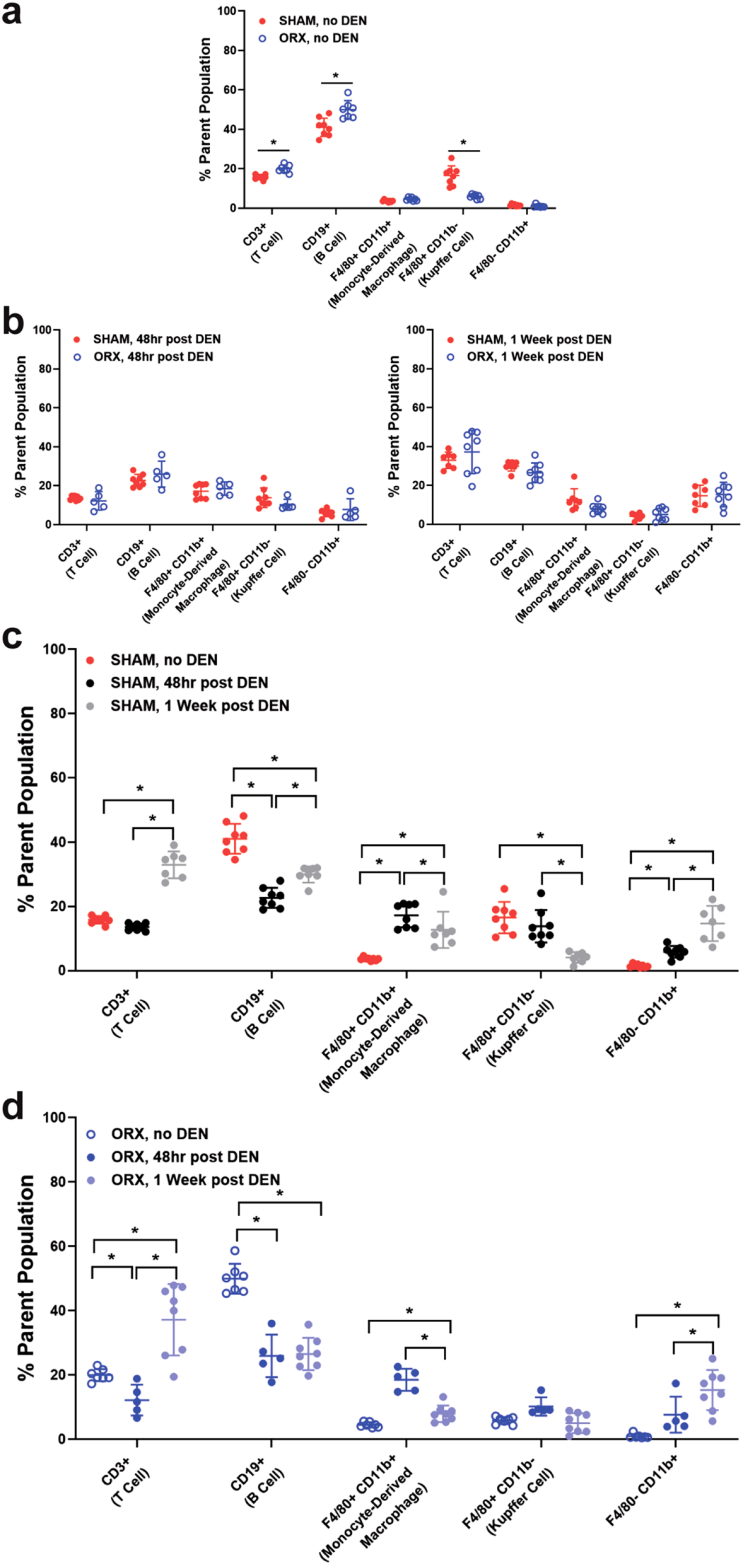
ORX- and DEN-mediated changes in immune cell recruitment to the liver. Liver immune cell populations were analyzed by flow cytometry as described in Materials and Methods and represented as percent of parent population and pre-gated on CD45+ and living cells. Positive cells were defined by fluorescence minus one (FMO) controls (See Supplementary Fig. S4 online for gating strategy). (**a**) ORX increased the proportions of CD3+ T cells and CD19+ B cells and decreased that of F4/80+ CD11b− Kupffer cells in VEH-treated mice (*p < 0.05, Tukey’s multiple comparisons). (**b**) DEN challenge in ORX mice did not alter immune cell proportions in comparison to SHAM mice. (**c**) DEN challenge in SHAM mice increased the percentage of CD3+ T cells at 1 week and decreased that of CD19+ B cells at 48 h and 1 week. The proportion of F4/80+ CD11b+ monocyte-derived resident liver macrophages increased at 48 h and subsequently decreased at 1 week following DEN, whereas that of F4/80+ CD11b− Kupffer cells decreased at 1 week after DEN. The percentage of F4/80− CD11b+ myeloid lineage cells increased at 48hrs and remained elevated at 1 week (*p < 0.05, Tukey’s multiple comparisons). (**d**) DEN challenge in ORX mice caused similar changes as DEN challenge in SHAM mice, except that the percentage of CD3+ T cells decreased at 48 h and that of F4/80+ CD11b− Kupffer cells did not change relative to baseline (*p < 0.05, Tukey’s multiple comparisons). All data represented as mean ± standard deviation.

To assess the influence of endogenous androgens on the makeup of hepatic immune cell populations, we measured lymphocyte subsets (CD3+ T cells and CD19+ B cells) and monocyte-derived liver macrophages in SHAM or ORX mice absent of DEN challenge and 2 and 7 days following DEN challenge (Fig. 5). Liver macrophages were defined using markers F4/80 and CD11b^32,33^ with F4/80+ CD11b+ and F4/80+ CD11b− cells defined as monocyte-derived resident liver macrophages and Kupffer Cells, respectively. Remaining F4/80− CD11b+ cells were defined as myeloid lineage cells including monocytes, granulocytes, and natural killer cells^34^. These immune cell populations were measured at 2 and 7 days following DEN challenge to capture the impact of sex hormone signaling on baseline hepatic immune cell composition as well as the potential impact of ORX-dependent differences in DEN metabolism on the acute immune response. Absent DEN challenge, ORX resulted in an increase in the percent of CD3+ T cells and CD19+ B cells, and a decrease in the proportion of F4/80+ CD11b− Kupffer cells (Fig. 5a).

Despite these baseline differences established by ORX, the proportions of immune cell subsets in the liver by 2 and 7 days after DEN challenge were essentially unaffected by castration (Fig. 5b). In both SHAM and ORX treatment groups, DEN resulted in an increased proportion of CD3+ T cells and a decreased percent of CD19+ B cells (Fig. 5c,d). Additionally, in both treatment groups, F4/80+ CD11b+ monocyte-derived liver macrophages and F4/80− CD11b+ myeloid lineage cells also increased. The percent of F4/80+ CD11b− Kupffer cells in SHAM mice decreased over time in response to DEN; however, there was no decrease observed in ORX mice (Fig. 5c,d, respectively), likely due a lower baseline proportion of this phenotype in ORX relative to SHAM mice (Fig. 5a).

## Discussion

Given the male predominance in disease incidence and mortality, building evidence of AR expression and activity, and an increasing number of available pharmacologic anti-androgens, the AR is a promising chemopreventive and therapeutic target in HCC. Pre-clinical models consistently indicate that *early* interventions, including castration and genetic knockout of AR, inhibit cancer onset and burden^3,9,35^, whereas later stage interventions exhibit significantly less anti-cancer activity^2–4,14^. The evidence collectively suggests that the optimal strategy for AR-targeted intervention is early in the hepatocellular carcinogenesis cascade.

What little is known about AR expression, activity, and suspected pro-carcinogenic mechanisms is limited to late-stage disease where targeted therapies are ineffective. The aim of our study was to determine the efficacy of early pharmacologic AR inhibition, interrogate mechanistic hypotheses potentially explaining how chemopreventive anti-androgen therapies inhibit hepatic carcinogenesis, and characterize AR expression and distribution in early carcinogenesis. To that end, we demonstrate: (1) Intervention with ENZ at 30 mg/kg indeed inhibits carcinogen-induced liver carcinogenesis; (2) Androgen deprivation alters hepatic metabolic zonation, reducing CYP2E1 expression and preventing activated DEN-induced toxicities including cell injury, death, and DNA-alkylation; and (3) Hepatic AR expression is upregulated during early DEN carcinogenesis and is largely due to AR expression within hepatic immune cells and fibroblasts with a minor contribution from hepatocytes (preneoplastic or otherwise).

DEN-induced hepatocellular carcinogenesis in rodents is a well characterized and commonly used model of HCC. Anti-androgen interventions occurring early in carcinogenesis consistently exhibit remarkable inhibition of HCC onset and burden in preclinical models^9,14,15^. Delayed interventions exhibit a time-dependent decrease in therapeutic efficacy^4^. Based on these studies, we hypothesized that chemopreventive intervention would improve the likelihood of observing a benefit to anti-androgen therapy in HCC. Our results support this hypothesis by identifying a statistically significant decrease in the number of pGST positive, pre-neoplastic hepatocytes following preventive ENZ treatment (*p* = 0.033, Fig. 1d).

To capture AR-dependent gene networks that inhibit DEN-mediated carcinogenesis, we employed an objective gene-expression microarray to identify the early, androgen-responsive hepatic transcriptome at the time of carcinogen challenge. We found no overlap in gene expression changes between preventive ENZ and ORX interventions (Supplementary Fig. S2 online), indicating that AR inhibition and ligand deprivation elicit functionally distinct transcriptional responses in liver.

The gene microarray yielded critical insight into potential mechanistic hypotheses explaining ENZ efficacy within our chosen model. We observed that DEN-challenged, ENZ-treated rats had uniform upregulated expression of lipid metabolism and cholesterol biosynthesis pathways, a metabolic pathway known to be tightly regulated to the periportal regions of the liver. Metabolic zonation relegates distinct metabolic processes to specific compartments of the larger hepatic lobule. The Wnt/β-catenin signaling pathway is a well characterized regulator of hepatic metabolic zonation. We hypothesized that preventive ENZ was modulating metabolic zonation and found consistent reduction of well-described hepatic β-catenin targets, Glul and CYP2E1 (Fig. 2c,d)^23,24^.

Within the context of chemopreventive anti-androgen therapies, DEN-mediated carcinogenesis is dependent on CYP2E1 bio-activation. DEN activation by CYP2E1 leads to the formation of reactive oxygen species causing oxidative damage and DNA adducts. Inhibition of the enzyme greatly reduces DEN’s ability to induce tumors^26,27^. In this study, we observed a decrease in CYP2E1 expression with DEN exposure. This reduction in expression is consistent with DEN pathogenesis. As CYP2E1 is required for DEN bioactivation, the centrilobular hepatocytes expressing CYP2E1 are those most susceptible to its toxic, bioactivated state. We attribute this DEN-induced reduction of enzyme expression to loss of CYP2E1 expressing hepatocytes. This observation is supported by an increase in detected ethyl-adducts and an increase in Cleaved Caspase III positive hepatocytes observed with DEN (Fig. 2d; Supplementary Fig. S2 online). Preventive, pharmacologic antagonism of the AR with ENZ caused a further reduction in CYP2E1 expression. This reduction occurs with a concurrent reduction in ethyl-adducts and CCIII positive centrilobular hepatocytes. These observations support the conclusion that ENZ inhibition of CYP2E1 in turn inhibits DEN bio-activation, reducing the production of DEN-induced reactive oxygen species and subsequent cell injury, DNA alkylation, and ultimately downstream carcinogenesis.

In spite of the success of early androgen deprivation therapy in rodent models of HCC, hepatic AR expression remains poorly characterized with analysis limited to late-stage disease. We characterized AR expression across multiple phases of DEN carcinogenesis from the early, acute phase 24 h after DEN challenge to established HCC tumors resulting from weeks of repeated DEN treatment. Given evidence of AR upregulation in hepatocyte-derived malignancy and the efficacy of early AR-axis targeted intervention, we hypothesized that the AR would be upregulated in early, pGST-positive initiated hepatocytes. We found that the hepatic AR is upregulated in the carcinogen-challenged liver (Fig. 3a). However, IHC clarified that the hepatic AR was within spindle and round cells in portal triads (Fig. 3b). Further interrogation identified these cells as co-expressing CD45 and SMA, suggesting that the cells were of leukocyte and activated fibroblast origin. Furthermore, the random, multifocal distribution of transformed, pGST-positive hepatocytes did not align with AR expression. We observed no appreciable differences in the distribution of AR signal with DEN challenge nor with anti-androgen interventions within 4 weeks of DEN challenge, suggesting changes in AR expression due to DEN is not a feature of early hepatocyte carcinogenesis.

Given our focus on early carcinogenesis and undetectable AR expression within hepatocytes, we wanted to confirm the presence of AR in DEN-induced rodent tumors. In our opinion, the only other study reporting similar results likely misinterprets normal hepatic structures (portal triads) as abnormal, AR-positive dysplastic foci, and adequate tissue assessment is obscured by abundant, non-specific immunoperoxidase staining artefact^9^. We observed AR-positive neoplastic hepatocytes in DEN-initiated tumors in a pattern (scattered, random) and cellular distribution (nuclear) consistent with human disease^36^. It is worth noting that AR-positive tumors represented a minority of those present. In total, these findings implicate an evolving role for the AR in hepatocellular carcinogenesis: an early, indirect role highlighted by the apparent upregulation in portal fibroblasts and leukocytes followed by an autonomous upregulation within the neoplastic hepatocyte. In agreement with our findings, an indirect role for AR in DEN carcinogenesis was also elegantly established by Kemp et al. who used *Tfm* mosaic neonatal mice to show that HCC is AR-dependent, yet autonomous hepatocyte AR expression is not a requirement for disease induction^15^. The same group later established that the sex hormonal effects on DEN carcinogenesis are dependent on wild-type levels of growth hormone^37^, which is well characterized to require intact hepatic androgen signaling^38^. Our discovery of non-hepatocyte, AR-positive cells raises the possibility for an intrahepatic, local and inflammatory influence of the AR. Furthermore, our finding that the pre-neoplastic hepatocyte is essentially devoid of AR expression suggests that genetic approaches ablating AR expression specifically in the hepatocyte may represent an inappropriate test of AR function in hepatocellular carcinogenesis that underestimates AR’s contribution^9^.

The liver is regarded as a critical immunologic organ^39,40^, and HCC is considered the end-stage event of chronic hepatic inflammatory processes. AR’s presence within immune cells suggests hormone signaling impacts inflammation associated liver carcinogenesis and may account for sexual dimorphism. Upon evaluation of AR+ cells within immune cell subsets from ORX and SHAM mice (Fig. 4), we observe AR expression primarily in DEN-challenged lymphocytes and monocytes. Despite ORX, the fraction of AR+ lymphocytes and AR+ monocytes was equally increased in both SHAM and ORX mice in response to DEN. An increase in AR+ immune cells after DEN injury is in line with previous studies implicating a role for androgens in increased liver inflammation following acute liver injury by carbon tetrachloride or DEN^41,42^. The apparent androgen-independent increase in AR+ immune cells subsequent to liver injury suggests a potential role for ligand-independent AR signaling within immune cells, similar to constitutively active AR signaling we detected in human HCC^43^ and is consistent with the previously highlighted importance of targeting the AR, as opposed to androgens, in the treatment of HCC^44^. Our future studies will aim to more precisely define the immune subsets which express AR and the AR signaling occurring within these populations.

The liver contains a very large population of macrophages, both resident and recruited, that are implicated in disease and therapeutic manipulation^45,46^. We observed a decrease in the proportion of F4/80+ CD11b− Kupffer cells in ORX mice (i.e. VEH treated) at baseline, strongly suggesting an androgen-dependent effect on resident Kupffer cell populations (Fig. 5). F4/80 and CD11b as well as CD68 expression classify mouse liver macrophage phenotypes. The resident monocyte- and embryologically-derived liver macrophages express F4/80, and may also express either CD11b, CD68, or both CD11b and CD68^33,34^. The renewal of Kupffer cells is debated, but evidence suggests that during homeostasis, Kupffer cells self-renew in the liver, and in the context of injury, monocyte-derived macrophages can infiltrate and differentiate into resident liver macrophages^46,47^. Our methods are unable to distinguish embryological-derived or monocyte-derived liver resident macrophages. However, we hypothesize that the percent of F4/80+ CD11b− macrophages was decreased by ORX and remained unchanged in response to DEN injury due to a lack of resident macrophage renewal. In agreement with our data, the importance of Kupffer cells in the sexual dimorphism of hepatocellular carcinogenesis has been previously reported^21^. The F4/80+ CD11b+ CD68− and F4/80+ CD11b− CD68+ liver macrophages possess distinct functions in response to DEN-mediated hepatocyte necrosis^33^. Future studies with more precise classification of liver macrophages in response to DEN and androgen manipulation may reveal functional differences attributable to sex hormones relevant to liver disease^45^.

Despite any differences in baseline immune cell proportions between SHAM and ORX mice, both groups had similar immune cell recruitment in response to DEN (Fig. 5). There were increased F4/80− CD11b+ cells, which include granulocytes, natural killer cells, and monocytes, representing an expected acute inflammation in response to DEN. The monocyte portion of these F4/80− CD11b+ cells may have acquired F4/80 surface expression to become monocyte-derived F4/80+ CD11b+ liver macrophages, explaining in part the rise in the percent of F4/80+ CD11b+ cells after DEN. Matching our data, Chen et al.^48^ found that the proportion of monocyte-derived F4/80+ CD11b+ liver macrophages was increased during chronic DEN exposure^48^. This rise in monocyte-derived F4/80+ CD11b+ macrophages was attenuated by deletion of GADD34, a protein upregulated in response to DNA damage, including DEN-mediated DNA adduct formation. Despite this link between signaling initiated in response to DNA damage and increased percent of F4/80+ CD11b+ liver macrophages, we did not find that reduced DNA adduct formation subsequent to AR-axis blockade led to decreased monocyte-derived F4/80+ CD11b+ liver macrophages. However, in this experiment, we did not chronically administer DEN, and with chronic exposure, small differences in immune cell recruitment in ORX relative to SHAM mice may be accentuated and become detectable. Regardless of the hormonal effects on DEN-induced DNA damage, consequent pathologic, chronic inflammation can drive the onset and progression of liver disease^39,49^. Based on the sexual dimorphism of autoimmune diseases, cancer, and specifically DEN carcinogenesis, androgen regulation of hepatic immune cells, including macrophage populations, and inflammation may contribute to liver disease onset or progression.

## Conclusions

**Figure 6:**
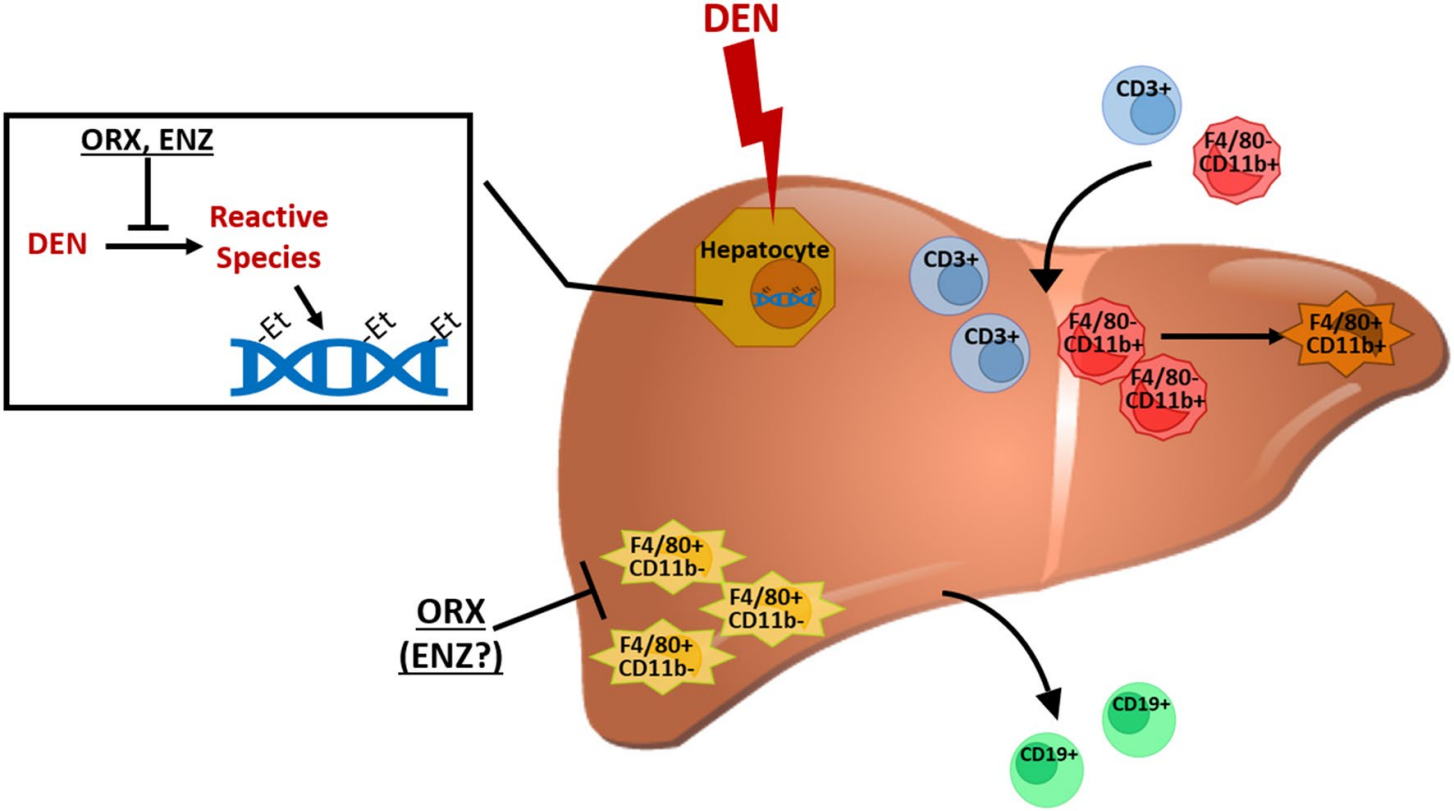
Graphical hypothesis of the impact of AR-signaling on DEN-mediated carcinogenesis. DEN is metabolized by CYP2E1 in hepatocytes into reactive species that create DNA ethyl adducts and induce liver damage. In the acute immune response to DEN, the proportions of CD3+ T cells, F4/80− CD11b+ myeloid lineage cells, and F4/80+ CD11b+ monocyte-derived resident liver macrophages are increased coinciding with a decreased proportion F4/80+ CD11b− Kupffer cells and CD19+ B cells. Inhibition of the AR/androgen signaling axis reduces DNA adduct formation in hepatocytes, decreases liver damage, and decreases baseline F4/80+ CD11b− Kupffer cells, yet does not alter relative immune cell recruitment. The image of the liver is from TogoTV (2016 DBCLS TogoTV / CC-BY-4.0), https://doi.org/10.7875/togopic.2014.7.

Our study enhances our understanding of androgen signaling in liver carcinogenesis and provides some novel mechanistic insights. Androgen signaling plays a key role in early stages of hepatocellular carcinogenesis, response to carcinogens, and the inflammatory processes driving the cancer cascade. We identify a novel role for androgenic promotion of liver carcinogenesis through increased DNA-alkylation, perhaps mediated by CYP2E1. Androgens impact immune cell populations, including liver macrophages, T-cells, and B-cells that are relevant to the acute response to DEN-induced liver injury while also impacting the subsequent inflammatory processes driving the carcinogenesis cascade (Fig. 6).

Our work provides insight into male susceptibility to HCC. Androgens may impact response to DNA-alkylation and mutagenic events, baseline hepatic immune composition, and AR-mediated immune signaling in the subsequent immune response (Fig. 6). Our studies provide additional support for the targeting of the AR for chemoprevention in high-risk individuals but highlight the critical need to better understand complex AR liver biology in multiple tissue compartments to inform the rational design of future studies.

## Materials and methods

Please see Supplementary Methods online for more common benchtop methods including qRT-PCR analysis and protein extraction and immunoblotting.

### Generation of biosamples

All murine experiments were conducted under an Institutional Animal Care and Use Committee (IACUC) protocol (2015A00000079) approved by the IACUC at The Ohio State University (Columbus, OH) in compliance with their Animal Care and Use Program. Six-week old, male Sprague Dawley rats (Envigo, Indianapolis, IN) were stratified by weight into treatment groups (n = 6) upon reception. Animals were surgically (ORX) or sham (SHAM) castrated (age = 7 wks) based on group assignment one week prior to initiating treatment. Treatment consisted of SHAM rats receiving 10, 30, or 100 mg/kg oral ENZ (Xtandi, enzalutamide, Astellas, Tokyo, Japan) diluted in 300 g/mol polyethylene glycol 300 (PEG 300; Sigma-Aldrich, St. Louis, MO, 807484) once per day. Treatment started two weeks prior to carcinogen challenge (age = 8 weeks) and lasted to sacrifice (SAC). PEG300 vehicle (VEH) was delivered to control groups with ORX rats serving as a low-testosterone control and SHAM animals serving as negative controls. We initiated carcinogenesis with 50 mg/kg intraperitoneal (IP) DEN (Sigma-Aldrich, 73,861) (age = 10 wks) followed 24 h later by two-thirds PH followed again by IP DEN of the same dose (Fig. 1a; See Supplementary Methods online for PH procedure). Liver samples were collected at PH and SAC, flash frozen and stored at − 80 °C until prepared and utilized for molecular analysis. Cross-sections of liver were formalin-fixed, routinely processed and paraffin-embedded (FFPE) and sectioned for routine and immunohistochemical (IHC) staining.

A parallel study for gene expression analysis was performed with a near-identical design using only 30 mg/kg dose of ENZ as treatment group and adding a PH-only, no carcinogen challenge, VEH-treated control group (n = 4; Fig. 2a). Liver samples were stored in RNAlater (Sigma-Aldrich, R0901) according to the vendor’s protocol.

To characterize AR expression throughout all stages of DEN carcinogenesis, SHAM rats receiving VEH were subjected to the DEN/PH/DEN challenge (n = 8). Animals recovered for 4-weeks then were given weekly 50 mg/kg IP DEN injections for 6, 10 or 14 weeks to accelerate tumorigenesis (Supplementary Fig. S3 online).

To characterize the liver-resident immune cell composition following ORX and DEN challenge, 8-week old male C57BL/6 J mice (Jackson Laboratory, Bar Harbor, ME) were ORX or SHAM castrated two weeks prior to challenge with VEH or 50 mg/kg IP DEN (n = 8/group). DEN-treated mice from ORX and SHAM treatment groups were sacrificed at 48 h and 1 week following DEN injection. VEH mice were sacrificed 1 week following VEH injection (Fig. 4a). The right lobes of liver were collected and processed for flow cytometry.

### Histology, immunohistochemistry, and image analysis

Haematoxylin & eosin (H&E) and IHC staining were performed on 4-µm thick FFPE tissue sections. Hepatic lesions were classified according to INHAND Hepatobiliary guidelines^50^. See Supplementary Table S1 online for details for IHC reagents used, experimental conditions, and brief protocols. In the first study (n = 6 per group), pGST immunoperoxidase positive foci were counted to assess carcinogenic outcomes. Briefly, a blinded observer took ten representative 40 × total magnification photomicrographs of each liver. Images were uploaded into Image Pro Plus v7.0 (Media Cybernetics, Rockville, MD) where an algorithm to quantify pGST-positive foci was generated using the Count/Size feature. A pGST-positive focus included any hepatocyte or cluster of hepatocytes exhibiting immunoperoxidase-positive staining. In the parallel study focusing on gene expression (n = 4 per group), CYP2E1 peroxidase positive staining area surrounding central veins was measured using Olympus cellSens Standard software about five 200 × total magnification fields. Glutamine synthetase (Glul)-positive peroxidase staining was quantified using the Positive Pixel Count Algorithm in Aperio’s Scanscope software (Leica Biosystems Inc., Buffalo Grove, IL) on a 20 × low-power magnification image for available liver samples. To assess DEN mediated hepatocyte death, we evaluated hepatocytes with immunoperoxidase positive, cytoplasmic staining for Cleaved Caspase III across five 400 × high-powered fields focused on central veins. Each of these stains were performed on a subset of animals (n = 3) and one tissue in the VEH/VEH was lost during processing, resulting in n = 2 for this group.

### Microarray analysis

Microarray analysis was performed at The James Comprehensive Cancer Center’s Genomics Shared Resource. RNA was extracted from liver tissue with the Animal Tissue RNA Purification Kit (Norgen Biotek, Thorold, Ontario, Canada; #25,700). Briefly, RNA was hybridized to GeneChip Rat Gene 2.0 ST Arrays. Probe signal was background corrected, normalized and non-changing and/or undetected probes removed. Differentially expressed genes (DEGs) were determined (limma) using an FDR-corrected p-value of < 0.2. Significantly different genes were visualized using pheatmap, using Manhatten clustering, and the treatment groups indicated.

### DNA preparation and liquid chromatography-mass spectrometry (LCMS) adduct assay

DNA was extracted from rat livers using the DNeasy Blood and Tissue Kit (Qiagen, 69504). DNA was processed and the adduct *O*^2^-ethyl-thymidine was quantified using methods similar to those previously published^51–53^. Briefly, approximately 5 µg of extracted DNA was hydrolysed by sequential enzymatic reactions with nuclease P1 (USBiological, Salem, MA; N7000), phosphodiesterase I (Sigma-Aldrich, P3243), and alkaline phosphatase (ThermoFisher, EF0651). Hydrolysed *O*^2^-ethyl-thymidine was purified and concentrated using an Oasis HLB cartridge (Waters, Milford, MA; WAT094225) and then quantified via LC–MS/MS tandem mass spectrometry using Waters Quattro Premier equipped with UPLC liquid chromatography and tandem mass spectrometry. LC conditions included mobile phases of A (10 mM ammonium formate/0.1% formic acid) and B (methanol/0.1% formic acid) with a gradient of 10–100% of mobile phase B which specifically 0.0 min (90% A and 10% B), 5.0 min (65% A and 35% B), 10.0 min (85% B), 18 min (100% B), 20 min (90% A and 10% B) at a constant flow rate of 200 µl/min. Analytes were separated using a C8(2) column (Phenomenex, Torrence, CA). The mass transition of the analyte following precursor/product ion pair of *m*/*z* was 271.2–155.0 for *O*^2^-ethyl-thymidine. The mass spectrometer was tuned to its optimal sensitivity by direct infusion of pure O^2^-ethyl-thymidine. The data acquisition and peak integration were performed using Mass Lynx software (Waters).

### Murine liver flow cytometry

See Supplementary Methods online for mouse liver preparation for flow cytometry. All data was acquired on a BD LSRFortessa within one week of sample collection and fixation. Bead-based compensation was performed for each experimental run. See Supplementary Fig. S4 online for the gating strategy. All gates were set on fluorescence-minus-one (FMO) controls prepared for each individual experiment. Data were analyzed with FlowJo software (version 10.5.3).

### Quantitative analysis

Statistical analyses were performed with Prism 8 (Graphpad, San Diego, CA). Figure legends contain corresponding test-specific analyses. Microarray analyses was undertaken using R platform for statistical computing and implementing various libraries (version 3.6.2)^54^.

## Supplementary Information

Supplementary Information.

## Data Availability

The datasets generated during and/or analysed during the current study are available from the corresponding author on reasonable request.
