# Supplementary material for: Inhibition of androgen/AR signaling inhibits diethylnitrosamine (DEN) induced tumour initiation and remodels liver immune cell networks
Source: Sci Rep. 2021 Feb 11;11:3646. doi: 10.1038/s41598-021-82252-x (PMC7878907; doi:10.1038/s41598-021-82252-x)
Supplement: Supplementary file 1 — Supplementary Information. [file 41598_2021_82252_MOESM1_ESM.pdf]

Inhibition of Androgen/AR Signaling Inhibits Diethylnitrosamine (DEN) Induced Tumour Initiation and Remodels Liver Immune Cell Networks

Timothy H. Helms[†,1,2], Riley D. Mullins[†,3], Jennifer M. Thomas-Ahner[2], Samuel K. Kulp[2,3], Moray J. Campbell[2,3,4], Fabienne Lucas[5], Nathan Schmidt[2], Dana M. LeMoine[6], Surafel Getaneh[3], Zhiliang Xie[3], Mitch A. Phelps[3], Steven K. Clinton[2,7], Christopher C. Coss[2,3]*

[1] Department of Veterinary Biosciences, The Ohio State University, Columbus, Ohio, 43210, USA.
[2] The Comprehensive Cancer Center, The Ohio State University, Columbus, Ohio, 43210, USA.
[3] Division of Pharmaceutics and Pharmacology, College of Pharmacy, The Ohio State University, Columbus, Ohio, 43210, USA.
[4] Biomedical Informatics Shared Resource, The Ohio State University, Columbus, Ohio, 43210, USA.
[5] Division of Hematology, Department of Medicine, The Ohio State University, Columbus, Ohio, 43210, USA.
[6] University Laboratory Animal Resources, The Ohio State University, Columbus, Ohio, 43210, USA.
[7] Division of Medical Oncology, Department of Internal Medicine, The Ohio State University, Columbus, Ohio, 43210, USA.

* To whom correspondence should be addressed. Tel: +1 614-688-1309; Fax: +1 614-292-5369; Email:

coss.16@osu.edu

† authors contributed equally to this work.

Supplementary Methods:

Rodent Care. All rodent studies were conducted according to protocols approved by The Ohio State University Institutional Animal Care and Use Committee. Rodent studies were group-housed under conditions of constant photoperiod (12-hour light/12-hour dark), temperature and humidity with *ad libitum* access to water and standard pelleted chow. Rats and mice were euthanized by CO_2 inhalation followed by exsanguination.

Partial Hepatectomy (PH). Rats were anesthetized with isoflurane (Henry Schein Inc., Melville, NY, 1182097) and placed in dorsal recumbency on a heated pad. After routine preparation of the surgical site, an abdominal incision was made and the left lateral and median lobes of liver were mobilised, ligated at the hilus with suture, and resected. After resection, each animal was administered 0.05 mg/kg buprenorphine (Reckitt Benckiser, Ltd, United Kingdom) subcutaneously. The abdominal wall and skin were each closed with absorbable suture, and animals recovered in a warmed cage with fresh bedding.

RNA extraction, cDNA conversion and qRT-PCR. RNA was extracted from liver tissue with the Animal Tissue RNA Purification Kit (Norgen Biotek, Thorold, Ontario; #25700). RNA concentration and purity were assessed using a Nanodrop Spectrophotometer (ThermoFisher, Waltham, MA; ND-1000). 2 μg of RNA was converted to cDNA with the High-Capacity cDNA Reverse Transcription Kit (ThermoFisher, 4368814). Converted cDNA was diluted 1:5 in RNAse free water. qPCR was performed on converted cDNA using PowerUp SYBR Green Master Mix (ThermoFisher, A25780) with the QuantStudio 7 Flex System (ThermoFisher). Statistical analyses were performed on ΔCT values using β-2 microglobulin as an in-house reference gene.

Protein extraction and Immunoblotting. Tissue lysates were produced with RIPA buffer (ThermoFisher, #89900) with added protease and phosphatase inhibitors (ThermoFisher, #78440). Equivalent tissue protein quantities, as determined by the Pierce BCA Protein Assay Kit (ThermoFisher, #23225), were loaded onto 4-12% SDS-PAGE gels (ThermoFisher, WG1403A), and lysate proteins were separated by size and charge via gel electrophoresis. Proteins were then transferred onto nitrocellulose membranes (Bio Rad, #1704271) using the Trans-Blot Turbo Transfer System (Bio-Rad, 1704150). Transfer quality was assessed with Ponceau S (Neta Scientific, #: P56200). Membranes were blocked with 5% nonfat-

dried milk (NFDM) in Tris-buffered saline containing 0.1% Tween 20 (TBST) for 1 hour, and then washed 3 additional times in TBST. Membranes were incubated at 4°C overnight with specific primary antibody diluted in 5% Bovine Serum Albumin in TBST: AR (Abcam, Cambridge, MA; ab133273) – 1:2000; CYP2E1 (Abcam, ab28146) – 1:5000; GAPDH (Cell Signaling, Danvers, MA; #5174) – 1:5000. They were then washed 3 times in TBST and then incubated with diluted goat Anti-Rabbit IgG-horseradish peroxidase conjugate secondary antibody (Sigma-Aldrich NA934V, 1:5000) in 1% NFDM in TBST at room temperature for 1 hour. Specific proteins were detected using enhanced chemiluminescence reagents.

Mouse liver extraction and Immunoblotting. Single cell suspensions were obtained from fresh liver samples using the MACS Liver Dissociation Kit as per manufacturer's protocol, including enzyme treatments, using the gentleMACS Octo Dissociator (Miltenyi Biotec, 130-095-937). The resulting suspension was filtered through a 70-µm cell strainer and washed in PBS. Red blood cells were removed with RBC Lysis Buffer (ThermoFisher, 00-4300). The cell suspension was again filtered through a 70-µm cell strainer and washed prior to staining. Approximately 1x10^6 to 3x10^6 total cells were resuspended in 100 µl per panel of LIVE/DEAD Fixable Near-IR Dead Cell Stain Kit (1:333, Invitrogen, L34976) diluted in PBS for 15 minutes at room temperature in the dark. After washing the samples in PBS with 1% BSA (FACS buffer), Fc receptors were blocked with PBS/5% rat serum for 5 minutes. Each sample was then aliquoted for surface (immune cell subset panel) and intracellular AR staining. Antibodies against cell surface proteins were added to the PBS/5% rat serum solution, and samples were incubated at 4°C for 30 minutes. The broad immune cell subset panel used FITC anti-CD19 (1:200, Biolegend, 152404), BV510 anti-CD3 (1:150, Biolegend, 100353), APC anti-CD45 (1:200, Biolegend, 103112) , BV450 anti-CD11b (1:270, Biolegend, 101251), and PE anti-F4/80 (1:33, Biolegend, 123110). The intracellular AR panel used APC anti-CD45 (1:200, Biolegend, 103112) and PE anti-AR (1:50, Santa Cruz, sc-7305). All antibodies were titrated for optimal performance. Samples were then washed and fixed in a solution of 1.7% formaldehyde (immune subset panels), or incubated in Foxp3 Fixation/Permeabilization (eBioscience, 00-5521-00) working solution for 18 hours at 4°C, washed, blocked in Permeabilization Buffer/2% rat serum, and then incubated at room temperature for 60 minutes with PE anti-AR (1:50, Santa Cruz, sc-7305). Samples were washed in Permeabilization Buffer and stored at 4°C until acquisition.

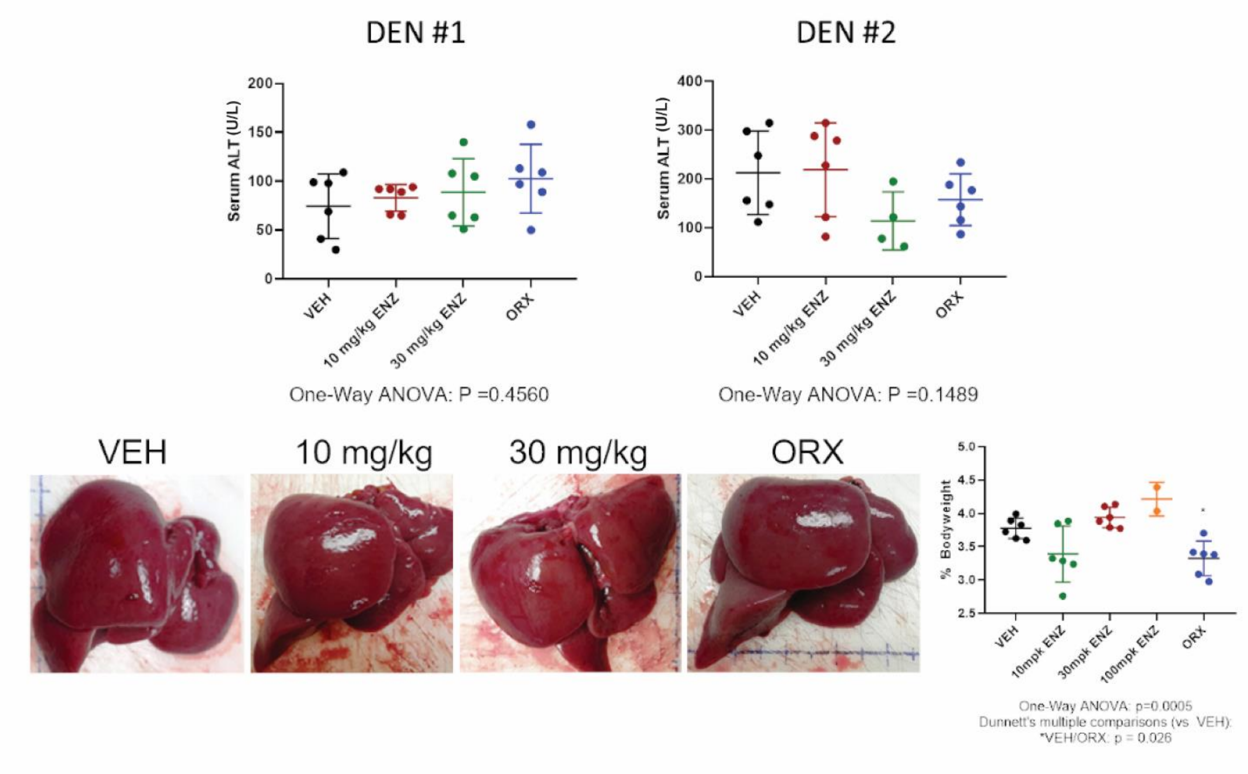

Supplementary Figure S1. Low dose ENZ is tolerated in the DEN/PH model of hepatocarcinogenesis. Top: Serum ALT (U/L) from animals collected 24 hours following DEN dose #1 (Left, at PH) or 24 hours following DEN dose #2 (right). Preventive anti-androgen therapies do not ameliorate acute hepatic necrosis 24 hours after each DEN challenge. p=0.456 for DEN #1 and p=0.1489 for DEN #2, one-way ANOVA. Bottom: There were no obvious differences between livers of different treatment groups as determined by gross appearance (left) and normalized liver weights (right). All data represented as mean ± standard deviation.

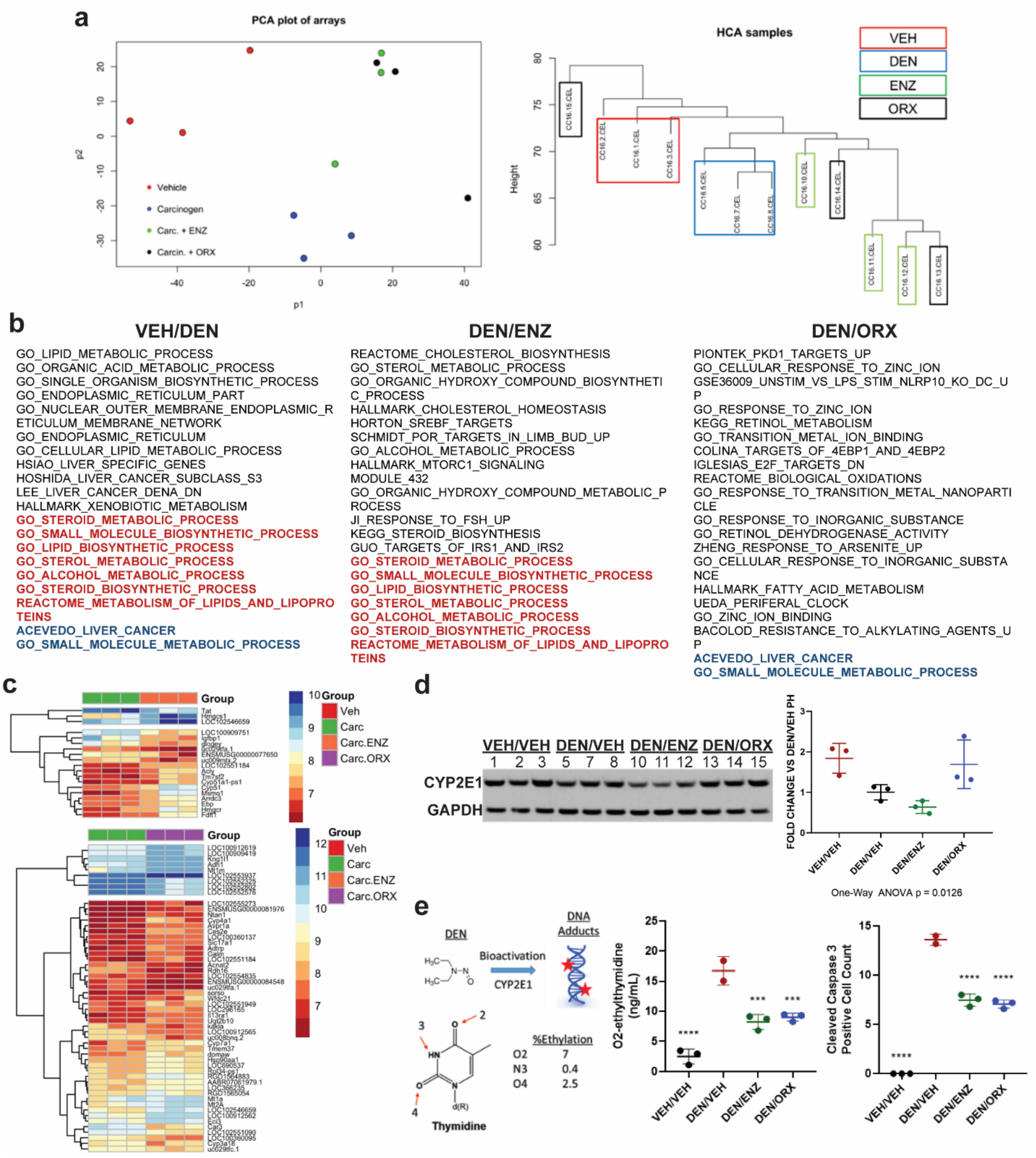

Supplementary Figure S2: Preventive anti-androgen treatments inhibit DEN carcinogenesis in a model specific manner. **(a)** Primary Component Analysis (PCA) and Hierarchical Clustering Analysis (HCA) identify treatment based segregation of mRNA microarray. Left - PCA of VEH (red), DEN (blue), ENZ (green), and ORX (black) treatment groups. Right - HCA of similar color scheme. Both highlight treatment based segregation mRNA expression patterns. **(b)** Gene set enrichment analysis (GSEA) of microarray in DEN challenged livers. 20 lowest p-value gene sets highlighted for each treatment cohort. Shared gene sets are bolded and highlighted in red (DEN/ENZ vs VEH/DEN) and blue (DEN/ORX vs

VEH/DEN). There are no overlapping gene sets between preventive anti-androgen treatment groups despite close hierarchical clustering seen in panel (a). **(c)** Left - Heatmap comparing DEN/VEH and DEN/ENZ (Carc vs. Carc.ENZ) and DEN/VEH and DEN/ORX (Carc vs Carc.ORX) treatment groups. **(d)** Western blot showing ENZ downregulation of CYP2E1 protein expression compared to GAPDH housekeeping control. Individual lanes are labeled with each sample's identification number, three samples per experimental group. Densitometry analysis – One-way ANOVA $p = 0.0126$. Full-length blots are presented in Supplementary Fig. 5. **(e)** Quantification of DEN adduct, O2-ethyl-thymidine, and Cleaved Caspase 3 positive foci. Left: Schematic demonstrating DEN adduct formation. Positional % ethylation data reported from Verna et al. (32). Middle: Preventive anti-androgen therapies inhibit DEN-mediated O2-ethyl-thymidine (ng/mL) DNA adducts. Right: ENZ and ORX treatment reduces the number of cleaved caspase positive foci surrounding DEN affected central veins (quantification of CC3 IHC). All quantitative analyses are One-way ANOVA with Dunnett's multiple comparisons (vs DEN/VEH): *** $p \leq 0.001$; *** $p \leq 0.0001$. All data represented as mean ± standard deviation.

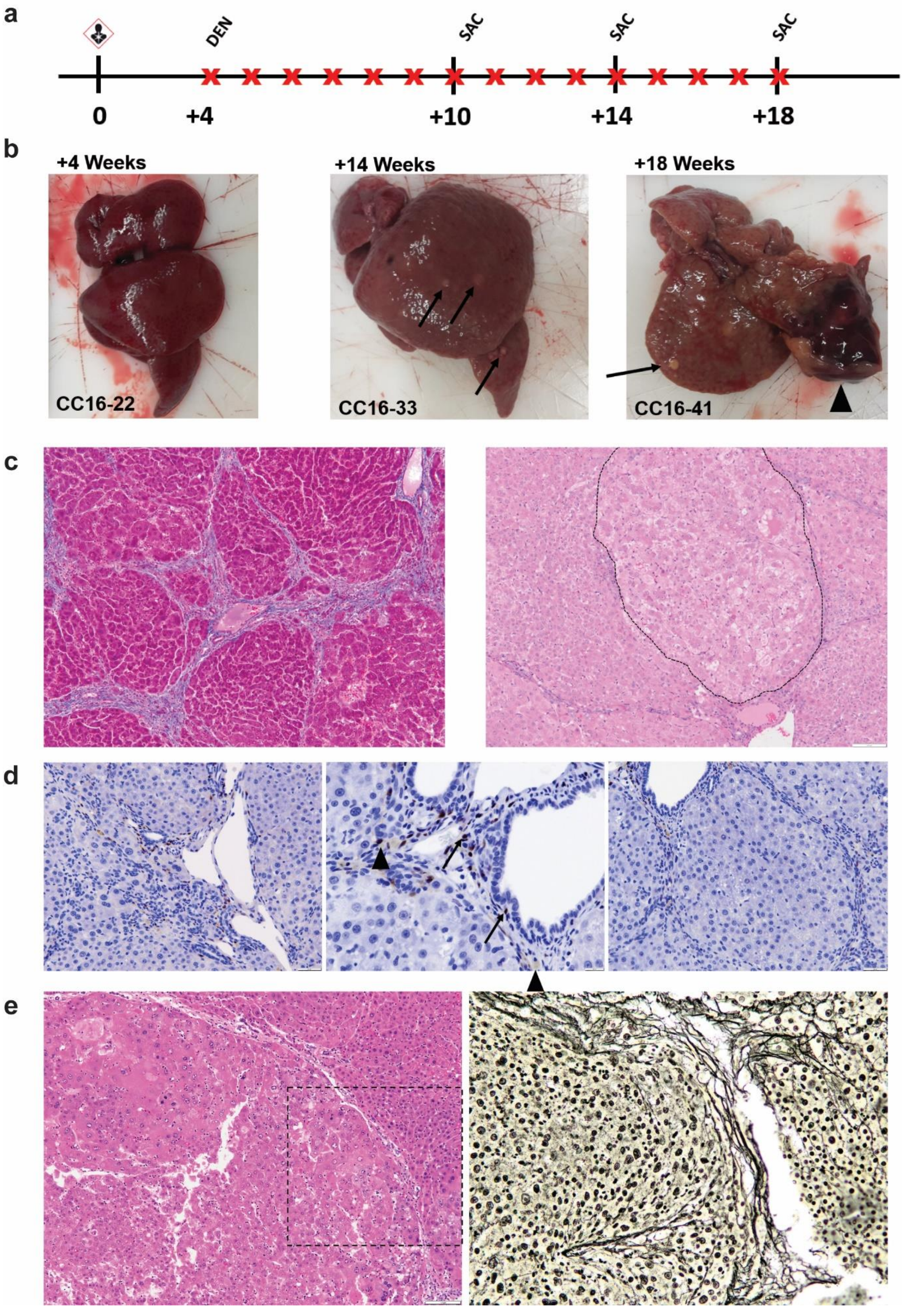
a
DEN
SAC
SAC
SAC
0
+4
+10
+14
+18
b
+4 Weeks
+14 Weeks
+18 Weeks
CC16-22
CC16-33
CC16-41
c
d
e

Supplementary Figure S3. Characterization of the hepatic AR in DEN-induced cirrhosis and tumors. **(a)** Simplified experimental design. SHAM-castrated, VEH-treated mice underwent the DEN/PH HCC induction protocol with weekly DEN injections (red *X*) administered for up to 18 weeks. Animals were sacrificed at 10, 14, and 18 weeks post-challenge. **(b)** Livers collected at necropsy exhibited progressive fibrosis and cirrhosis with nodule formation. Left – 4-weeks, mild capsular pitting consistent with early fibrosis; Middle – 14-weeks, livers are pale, fibrotic, with small nodules consistent with early adenomas (arrows); Right – severely fibrotic liver with more prominent adenomas (arrow) and large, tumorous coagulum of haemorrhage and necrosis (arrowhead). **(c)** Left – Masson's Trichrome (100x total magnification) of 18-week DEN-treated liver. Note the robust, bridging fibrosis (blue) with biliary hyperplasia. Right– Highlighted area shows a mixed eosinophilic and clear cell focus of hepatocellular alteration (100x total magnification). **(d)** IHC for AR in fibrotic livers (18 weeks after HCC induction). Left – AR expression remains restricted to the interstitium of the portal triad (200x total magnification). Middle – AR staining is nuclear (arrows). Light cytoplasmic staining (arrowheads) is non-specific as determined by rabbit IgG isotype, and no primary antibody controls (data not shown) (400x total magnification). Right – Regenerative nodules exhibit no parenchymal AR staining. Bounding fibrous connective tissue and ductular reaction exhibits some AR positivity (200x total magnification). **(e)** Left – H&E, photomicrograph of representative section showing DEN-induced HCC (occupies bottom left 2/3 of image). Right – Reticulin stain (Gordon and Sweet's), 400x total magnification of outlined region in left panel. Note loss of reticulin meshwork between neoplastic hepatocytes (left) compared to adjacent, compressed hepatocytes to the right of the fibrous bridge.

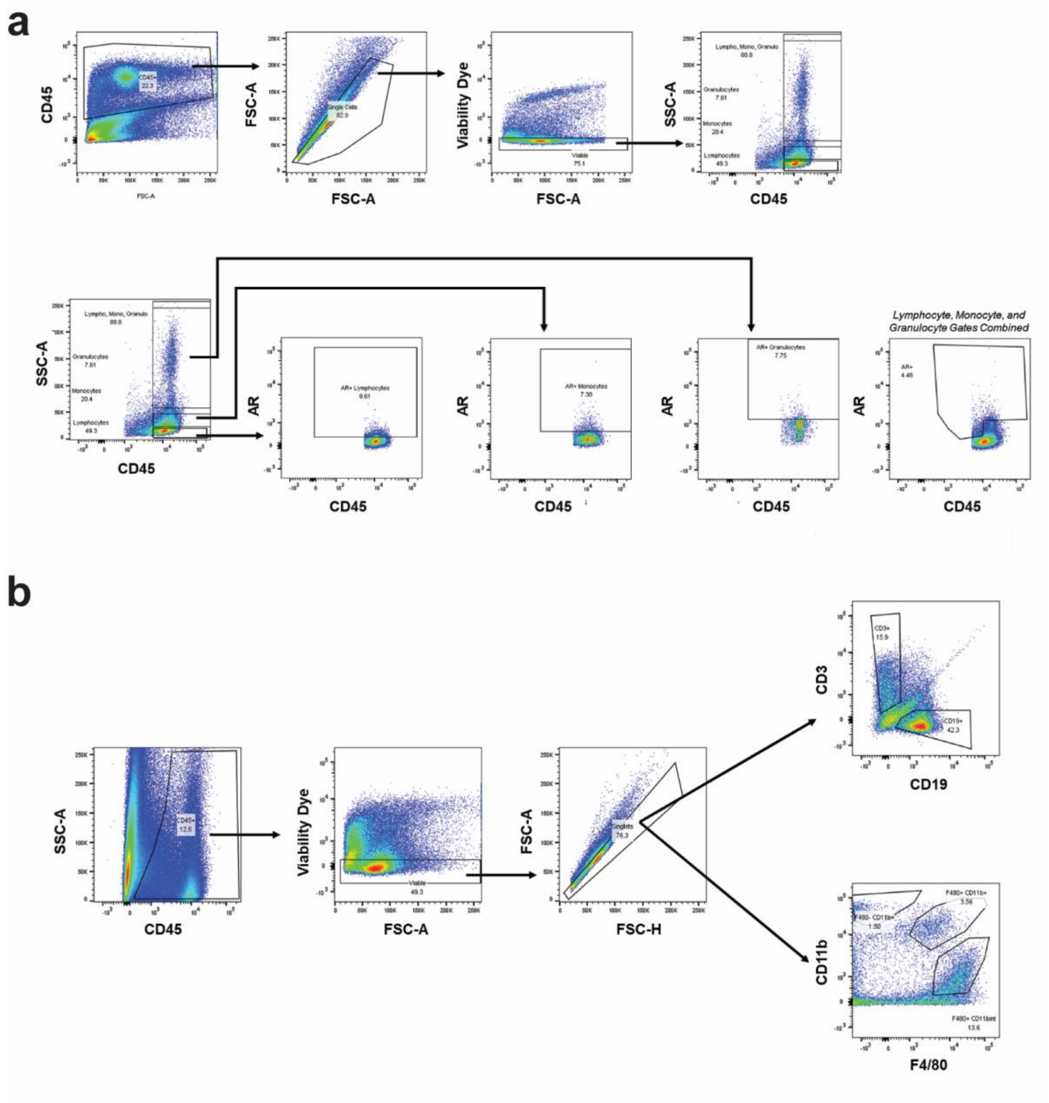

Supplementary Figure S4. Flow cytometry gating strategies. **(a)** Gating strategy used to produce Fig. 4. Cells were gated on CD45+, viable, single cells and subsequently parsed based on SSC properties into lymphocytes (SSC-low), monocytes (SSC-mid), and granulocytes (SSC-high). The total and each population were gated for AR+ cells. Gates were set based on FMO controls. **(b)** Gating strategy used to produce Fig. 5. Cells were gated on CD45+, viable, single cells. T cells (CD3+) and B cells (CD19+) were distinguished using a two-parameter plot with CD3 and CD19. Kupffer cells (F4/80+ CD11b-), monocyte derived macrophages (F4/80+ CD11b-), and myeloid cells (F4/80- CD11b+) were gated in a two-parameter plot of CD11b and F4/80. Gates were set based on FMO controls.

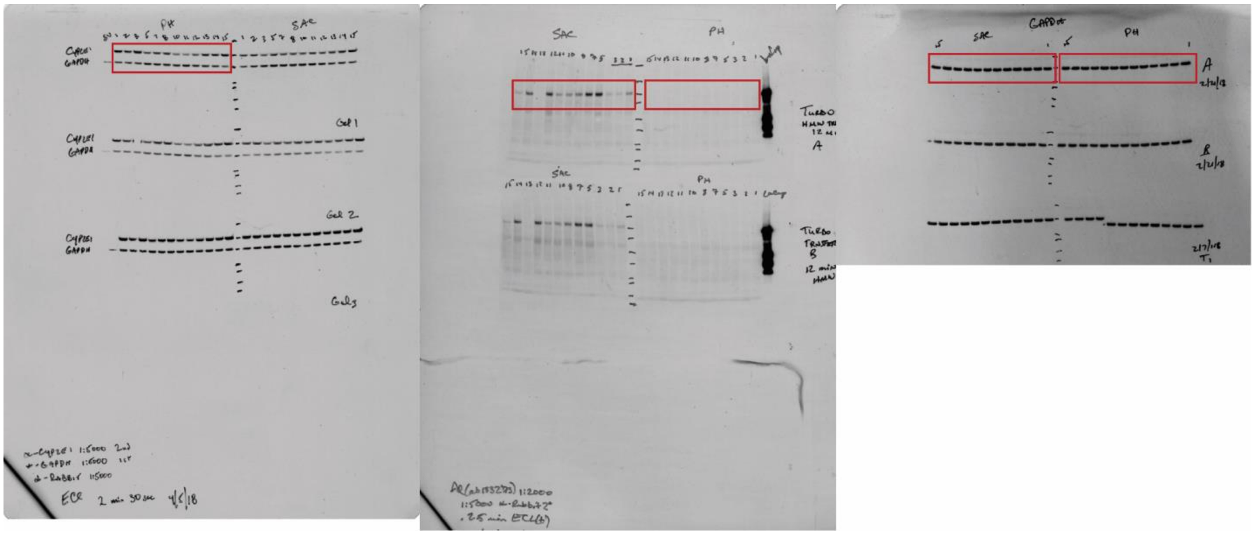

Supplementary Figure S5. Full-length, unedited Western blots. Western blots were cropped and rotated for presentation to ensure continuity in presentation order (i.e. VEH, DEN, ENZ, ORX). Left – Three technical triplicates of a Western blot for CYP2E1 and GAPDH. Lanes within the red box are shown in Supplementary Figure S2(d) with no changes in order. Middle – Two technical repeats of a Western blot for the Androgen Receptor (AR). Lanes within the red box were flipped about the horizontal axis in preparation of the Western blot shown in Fig. 3A. Right – Two technical repeats of a Western blot for GAPDH (top two). Lanes within the red box were flipped about the horizontal axis in preparation of the Western blot shown in Fig. 3a. The other Western blots are unrelated to this manuscript.

Supplementary Table S1. IHC reagents, experimental conditions, and protocol.

Target	Antibody	Antigen Retrieval	Protein Block	[Primary antibody]; incubation time	Secondary antibody; incubation time	Chromogen
**pGST**	MBL - MBL 311	Citrate (pH 6); heat (steam)	Power Block (Biogenex, HX0083); 10 minutes	1:1000; 1hr	HRP+Anti-Rabbit (Agilent, K4011); 30 min	DAB (Agilent, K4011); 10 minutes
**GluI**	Abcam - ab73593		Serum Free (Agilent; X0909); 10 minutes	1:2000; 1hr		
**Cleaved Caspase 3**	Cell Signaling - # 9661		Serum Free (Agilent; X0909); 10 minutes	1:180; 1 hr	Goat Anti-Rabbit IgG Antibody (H+L), Biotinylated (Vector, BA-1000) 1:500; 30 min	DAB (Vector, SK-4100)
**CYP2E1**	Abcam - ab28146			1:200; 1 hr	Goat Anti-Rabbit IgG Antibody (H+L), Biotinylated (Vector, BA-1000) 1:200; 30 min	
**AR**	Abcam - ab133273			1:250; 1hr		DAB (Vector, SK-4100)
**CD31**	Abcam - ab28364			1:50; 1hr		Vector Blue Substrate Kit, Alkaline Phosphatase (Vector, SK-5300)
**SMA**	Agilent - MO851			1:500; 1hr		
**CD45**	Abcam - ab10558			1:400; 1hr		

IHC for AR and Dual-Chromogen IHC were performed at The Ohio State University's Comparative Pathology & Mouse Phenotyping Shared Resource's Histology Lab.

4 micron thick FFPE tissue sections adhered to glass slides were deparaffinized in xylene and rehydrated in decreasing concentrations of ethanol to distilled water. Deparaffinized sections were submerged in citrate based (pH 6) antigen retrieval solution then heated in a vegetable steamer for 1 hour. Slides were washed in distilled water 3 times and sections were blocked with 3% peroxide in methanol for 15 minutes. Slides were again washed 3 times in distilled water then blocked with protein block for 10 minutes. Blocking solution was drained off, then the slide was incubated in diluted primary antibody for 1 hour at room temperature. Slides were rinsed 3 times with wash buffer then incubated with secondary antibody solution for 30 minutes at room temperature. Slides were again rinsed 3 times with wash buffer before incubating with chromogen for 5 minutes. Chromogen solution was rinsed and slides were counterstained with hematoxylin for 10-30 seconds.

Dual chromogen IHC procedures were performed sequentially starting with a second protein block and terminating with the second chromogen (Vector Blue) followed by rinsing in 0.5% ammonium hydroxide.

Slides were then dehydrated in increasing concentrations of ethanol and mounting solution and a coverslip was added to each.

Reagent Manufacturers: MBL – MBL International, Woburn, MA; Abcam – Cambridge, MA; Agilent – Santa Clara, CA; Biogenex – Freemont, CA; Vector – Burlingame, CA

Supplementary Table S2: Primers used for detection of cDNA converted, mRNA transcripts in rat.

Gene	Direction	5' to 3' sequence
AR (Full Length)	Forward	GGGGCAATTCGACCATATCTG
	Reverse	CCCTTTGGCGTAACCTCCCTT
B2M	Forward	CGTCACCTGGGACCGAGACA
	Reverse	TGAAGAAGATGGTGTGCTCATTGCT
CYP1A2	Forward	TCACAGGCGCCCTGTTCAAG
	Reverse	TGTGACTGTTTCAAATCCAGCTCCA
CYP2E1	Forward	GTGTTCACACTGCACCTTGG
	Reverse	CACCTCCTTGACAGCCTTGT
CYP7A1	Forward	CCTGCCGGTACTAGACAGCATC
	Reverse	GAAGTCCTCCTTAGCTGTGCGG
Glul	Forward	CCGCTCTGAACACCTTCCACC
	Reverse	CTTGCAGCGTAGCCCTTCCC
